# Anticancer Activity of Imidazolyl Gold(I/III) Compounds in Non-Small Cell Lung Cancer Cell Lines

**DOI:** 10.3390/ph17091133

**Published:** 2024-08-28

**Authors:** Rossana Galassi, Nicola Sargentoni, Sofia Renzi, Lorenzo Luciani, Caterina Bartolacci, Prasad Pattabhi, Cristina Andreani, Stefania Pucciarelli

**Affiliations:** 1 Chemistry Division, School of Science and Technology, University of Camerino, ChIP Via Madonna delle Carceri, 62032 Camerino, Italy; nicola.sargentoni@unicam.it (N.S.); lorenzo.luciani@unicam.it (L.L.); 2 School of Biosciences and Veterinary Medicine, University of Camerino, Via Gentile III da Varano, 62032 Camerino, Italy; sofia.renzi@unicam.it (S.R.); stefania.pucciarelli@unicam.it (S.P.); 3 University of Cincinnati College of Medicine, 3125 Eden Avenue, Cincinnati, OH 45219, USA; bartolcn@ucmail.uc.edu (C.B.); pattabpd@ucmail.uc.edu (P.P.); andreaca@ucmail.uc.edu (C.A.)

**Keywords:** metal-based anticancer, KRAS mutant cells, lung cancer, gold carbene complexes, gold phosphane compounds, gold(I) and gold(III), thioredoxin reductase, dihydrofolate reductase

## Abstract

Lung cancer is a leading cause of cancer-related death worldwide that needs updated therapies to contrast both the serious side effects and the occurrence of drug resistance. A panel of non-small cell lung cancer (NSCLC) cells were herein employed as cancer models. Eight structurally related gold(I) and gold(III) complexes with NHC and halides or triphenylphosphane ligands were investigated as lung cancer cell growth inhibitors. As expected, gold compounds with PPh_3_ were found to be more cytotoxic than homoleptic [(NHC)_2_-Au(I)]X or heteroleptic NHC-Au(I)X or NHC-Au(III)X_3_ complexes. Mixed ligand gold(I) compounds exhibiting the linear NHC-AuPPh_3_ (compound **7**) or the trigonal NHC-Au(Cl)PPh_3_ (compound **8**) arrangements at the central metal were found to be the best lung cancer cytotoxic compounds. Analysis of the TrxR residual activity of the treated cells revealed that these compounds efficiently inhibit the most accredited molecular target for gold compounds, the TrxR, with compound **8** reaching more than 80% activity reduction in lung cells. Some of the current cancer lung therapy protocols consist of specific lung cancer cell cytotoxic agents combined with antifolate drugs; interestingly, the herein gold compounds are both TrxR and antifolate inhibitors. The human DHFR was inhibited with IC_50_ ranging between 10–21 µM, depending on substrate concentrations, proceeding by a likely allosteric mechanism only for compound **8**.

## 1. Introduction

The search for anticancer drugs active against tumors with rather dramatic prognosis [1] or displaying a degree of selectivity toward healthy versus sick cells is an ongoing challenge [2]. Lung cancer is the leading cause of cancer-related death worldwide [3,4]. Recently, the development and approval of effective inhibitors against the oncogene *KRAS* have offered hope for lung cancer patients with these mutations [5,6]. For patients with advanced and or unresectable non-small cell lung cancer (NSCLC) who do not fit an approved molecular targeted therapy (e.g., *KRAS* wild-type), treatment options include regimens of radiotherapy and platinum-based chemotherapy, with or without immune therapy [4]. However, while only a subset of patients responds to these treatment regimens, the majority of them experience severe toxicity due to the off-target effects of these therapies. In this context, gold(I/III) complexes were originally studied as potential alternatives to the anticancer drug cisplatin, for example, in colon cancers [7], ovarian cancers [8], and lung cancers [9,10]. We have previously investigated the anticancer activity of some heteroleptic azolate/halide or imidazolate/phosphane gold(I) complexes. In particular, we found that depending on the lipo/hydro balance in the structure, imidazolate or pyrazolate gold(I) phosphane complexes exhibit the highest cytotoxic effect in a panel of cancer cell lines (including lung cancer) and the lowest off-target effects if compared to cisplatin [11,12]. Remarkably, imidazolyl-based NHC-AuX (with X = halide, NHC, or phosphane) compounds seem to be promising as gold-based anticancer drugs [9,13,14,15,16]. However, the relationship between the structure and the activity of these compounds, displaying similarity to the “backbone”, featured by the opposite head-to-tail polarity of the clinically available gold-based drug Auranofin [17], has not been fully explored yet. Furthermore, the effects of the oxidation at the central gold atom by halogen addition in an NHCAuX homolog series, as well as the electronic effects on anionic versus neutral 2-imidazolyl-2yl ligands have never been investigated in lung cancer cells. Stemming from our previous studies, in this work, we report the synthesis and characterization of 2-imidazolyl-2yl with 1,3-dimethyl or 1-benzyl-3-methyl substituents gold(I) and gold(III) complexes, with PPh_3_ or halides as co-ligands. Using panels of human NSCLC cell lung cancer cell lines, we established their overall stability, in vitro anticancer activity, and the structure–function relationship. In earlier works, DNA was considered to be the target for gold compounds [18,19], and lastly, even miRNA was found to be involved in the activity of a bis(diethyldithiocarbamate-gold(I))-bis(diphenylphosphine)methane, exhibiting lung cell cytotoxicity by altering the miRNAs expression profile in A549 lung cancer cells [20].

Even though some studies have reported a low target specificity for certain anticancer gold complexes [15,21,22,23,24], it has been largely demonstrated that selenoproteins such as TrxR are the prevalent molecular targets and play a fundamental role in the mechanisms of action of this class of compounds [11,15,18,19]. Here, we confirm that TrxR is a recognized molecular target of our gold-based drugs in NSCLC cell lines. Furthermore, by considering that antifolate drugs are currently used for lung cancer treatment, the most potent gold compounds were also investigated as inhibitors of the recombinant human dihydrofolate reductase (*h*DHFR), an NADPH—dependent enzyme responsible for the conversion of dihydrofolate (H_2_F) to tetrahydrofolate (H_4_F), which is essential for DNA biosynthesis and cell proliferation [25]. DHFR inhibitors, such as the “classical” antifolates methotrexate (MTX) and pemetrexed (MTA, approved by the FDA in 2008 for the treatment of non-squamous NSCLC patients) have been effective in treating many cancer types [26,27,28], their use is diminished as an effect of the high toxicity, the impaired cellular uptake and other resistance-associated mechanisms [29]. Alternative approaches have been used in the development of non-classical antifolates, which, thanks to a more lipophilic structure, can enter the cancer cell by passive transport, avoiding interaction with folate receptor (FR) transport systems [30]. Hence, to explore whether gold compounds may fall in this category and to ascertain eventual multiple mechanisms of action for the herein reported gold compounds, alongside the well-known TrxR, the ubiquitous human DHFR, that is not a selenoenzyme and is a key catalyst for the cell proliferation, was assayed in vitro.

## 2. Results and Discussion

### 2.1. Synthesis of the Gold(I/III) Compounds

**Scheme 1:**
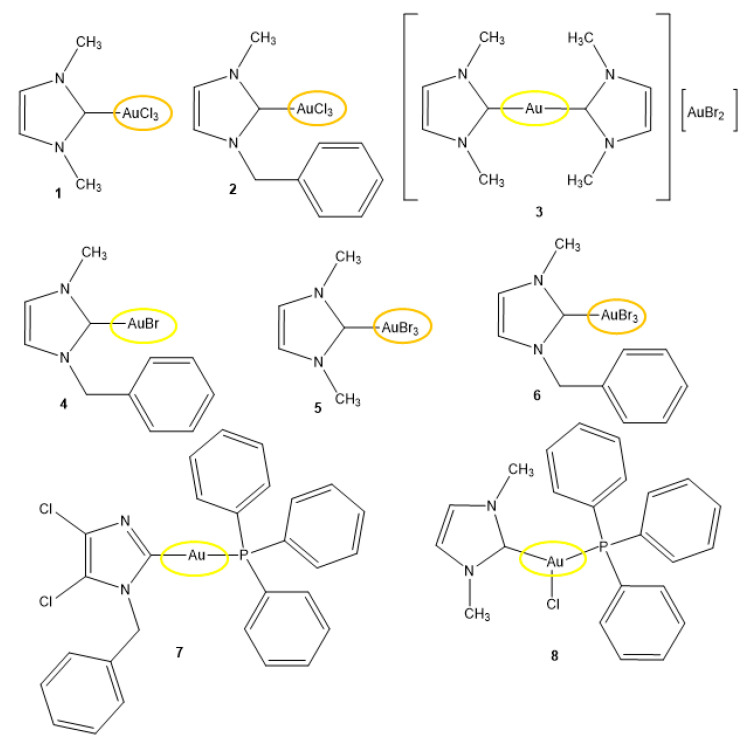
Sketch of the molecular structures for compounds **1**–**8** tested in this study.

**Figure 1:**
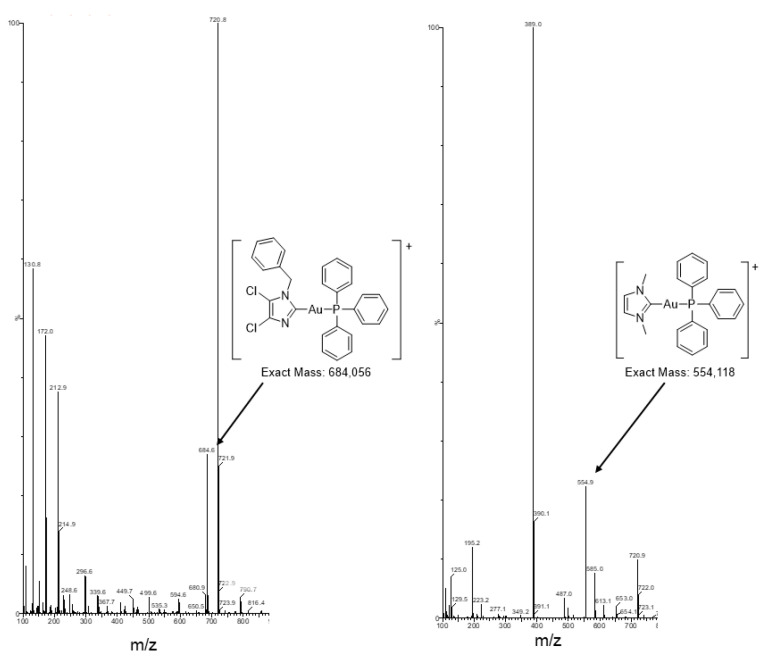
ESI MS spectra recorded for CH_3_CN solutions of compounds **7** and **8** in the range of 100–800 *m*/*z*. Both spectra display the peak at 720 *m*/*z* due to [(PPh_3_)_2_Au]^+^ as the main peak in addition to parent peaks.

**Figure 2:**
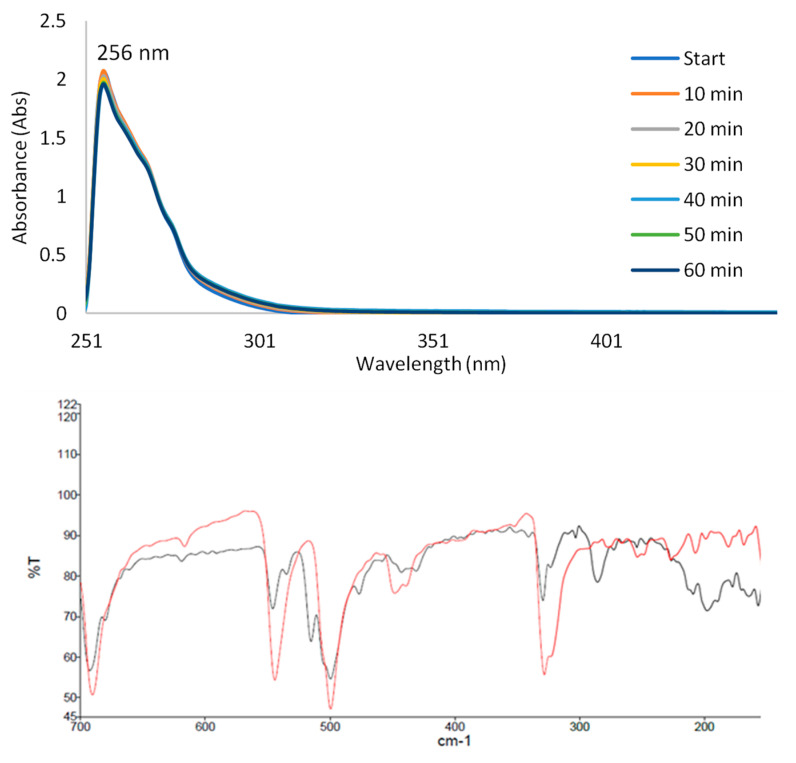
(**top**) UV−Visible spectra acquired for a DMSO solution of compound **8** over an hour; (**bottom**) overlayed FIR spectra of **8** (black curve) and PPh_3_AuCl (red curve, reported as a reference compound for the AuCl vibrational modes).

All the gold(I/III) herein studied contain structural similarities such as the presence of 2-imidazolyl-2yl (featured by symmetric or unsymmetrical substituents, namely as 1,3-dimethyl-2-imidazolyl-2yl, compounds **1**, **3**, and **5**, or 1-benzyl-3-methyl-2-imidazolyl-2yl, compounds **2**, **4**, and **6**) as NHC carbene ligands or as 1-benzyl-4,5-dichloro-2-imidazolyl-2-yl as anion ligand (compound **7**). Additionally, Au(I) compounds **7** and **8** contain the triphenylphosphine as co-ligand. Compounds **3**, **4**, and **7**, **8** are neutral, while compound **3** is cationic [31,32]. A view of the molecular structures of the compounds is reported in Scheme 1. The nature of these compounds was verified by IR, UV, ^1^H, and eventual ^31^P NMR spectroscopies by elemental analysis and ESI-MS spectrometry. Au(III) compounds were obtained by addition reactions of elemental bromine or PhICl_2_ to the corresponding linear NHC-Au-X starting compounds (with X = Cl or Br) [31,32]. The compounds were all characterized by analytical and spectroscopic techniques, and the molecular structure of most of the compounds herein applied were characterized by single-crystal X-ray diffraction; the mono-NHC or bis-NHC backbone of the compounds in solid state for compounds **3** and **4**, respectively, was revealed by X-ray structural determinations [31]. The ^1^H NMR spectra and the ESI-MS spectra recorded for DMSO solutions are reported in Supplementary Materials as Figures S2–S29. The molecular structures of compounds **7** and **8** are discussed in terms of spectroscopic and analytical data; for example, they exhibited the Im-AuPPh_3_ fragments in (+)ESI-MS spectra at 554 *m*/*z* and 684 *m*/*z*, respectively, in addition to the [(PPh_3_)_2_Au]^+^ ions at 720 *m*/*z* (Figure 1). Moreover, for compound **8**, the persistence of the AuCl stretching mode in the Far-IR spectrum at 329 cm^−1^ (with the shoulder at 323 cm^−1^) beside the least featuring Au-P stretching mode at 197 cm^−1^) [33] sustained the final attribution of a trigonal geometry around the metal center in the solid state (Figure 2, bottom). In DMSO-d^6^ solutions, the ^31^P NMR spectra of compounds **7** and **8** displayed singlets at about 42 and 32 ppm, highlighting the different electronic environments around the ^31^P nuclei, respectively.

#### Solution Stability Studies

Stability studies were led by acquiring UV−visible spectra of 100 µM DMSO or methanol solutions of compounds **1**∓**8** over time (Figures S19–S29 in Supplementary Materials). The UV-visible spectra of compounds **1**∓**8** displayed intense absorptions in the range 255–257 nm attributable to imidazolyl ligands, and broad bands around 290–336 nm for the AuX_3_ moieties absorptions. The UV visible spectra of compounds **7** and **8** recorded in DMSO or CH_3_OH solutions showed continuous absorptions from 210 to 290 nm. In general, the compounds with the C-Au-P chemical environment were more stable than those with C-Au-X or C-AuX_3_ chemical environments (with X = Cl or Br). The stability of NHC-AuX_3_ complexes (with X = Cl, compounds **1** and **2**, with X = Br, compounds **5** and **6**) was affected by the nature of both the substituents on the imidazolyl ligand and of the halide, showing a small decrease of the UV bands maxima in an hour. By analyzing the spectral data of compounds **1** and **2**, a percent concentration reduction of about 4–5% was estimated within an hour, grading them eligible for biological tests. The partial instability may be attributed to the formation of cationic bis-carbeneAuCl_2_ species (Equation (1)), with the [bis-carbeneAuX_2_]^+^ and the respective AuX_2_^−^; these ions were actually detected as positive and negative ions in the ESI-MS spectra (see Figures S12 and S13). Compounds **5** and **6** are the least stable in solution with spectral changes within an hour due not only to mono-/bis-carbene conversion but to likely additional interactions with DMSO, as discussed in the literature [34]. Additionally, the (+)ESI MS spectrum of the acetonitrile solution of compound **6** is particularly rich in positive ions (Figure S16). As concerns the mono-/bis-carbene formation, it is likely initiated by the large trans effect of the carbene ligands, which induces the chemical lability on the trans halide, triggers cell lines, the formation of bis-carbeneAu(III) species, and AuX_2_^−^ or AuX_4_^−^ anions [35,36,37]:

2[NHC-AuX_3_] ⇔ [[(NHC)_2_AuX_2_] [AuX_2_^−^] + 2X (1)

Evidence of the formation of cationic bis-carbeneAu(III) species was detected for compounds **1**, **2**, **5**, and **6** in (+)ESI-MS spectra, while the corresponding counterions were observed in the (−)ESI-MS (Figure S13). Additionally, the cationic bis-carbene-Au(I) complex **3** was more stable than the analog monocarbene-Au(I)Br species **4**, likely due to a stabilization effect of the phenyl group in the imidazolyl moiety that is not applicable in compound **5**.

Of note, compounds **7** and **8**, displaying the C-Au-P chemical environment, exhibited the highest stability in solution, with the former a bit more stable than the latter in DMSO, in methanol, and in PBS buffered solutions (Figure 2 and Figures S25–S29 in Supplementary Materials).

### 2.2. The Au(I) Compounds with the C-Au-P Environment Are the Most Cytotoxic

**Figure 3:**
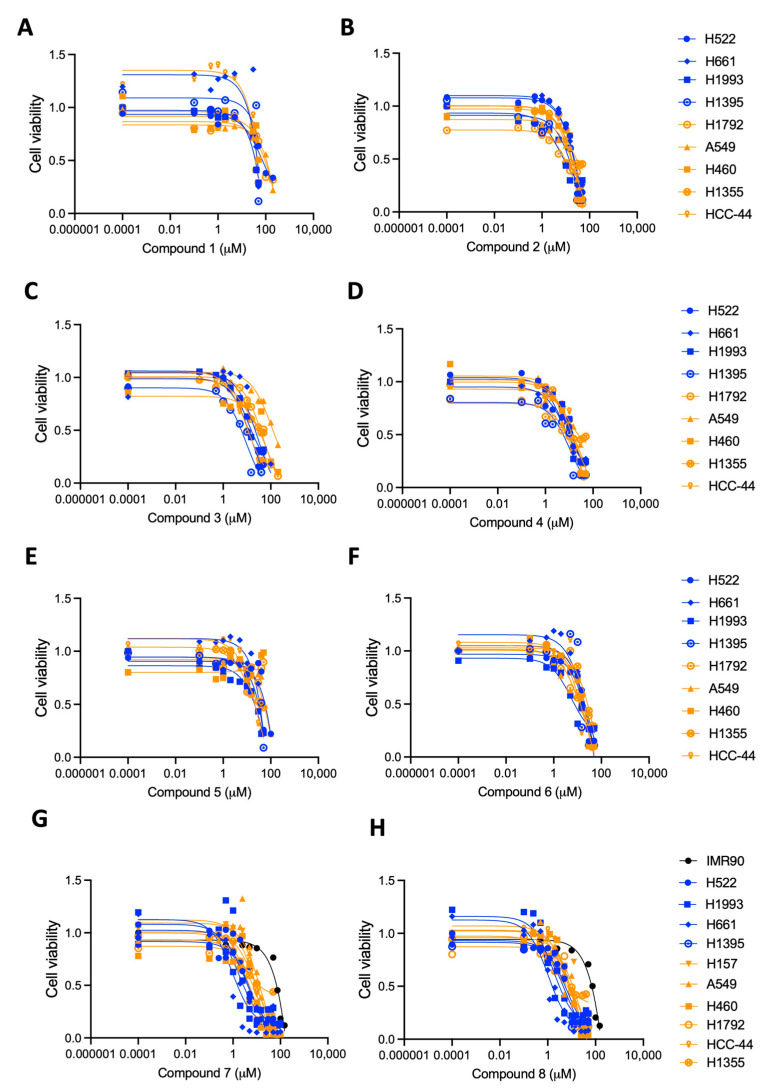
Au(I) compounds with the C-Au-Br environment are the most potent anticancers**.** (**A**–**H**): MTT viability assays of the NSCLC cell line treated with increasing concentrations of the gold compounds **1**–**8** for 48 h. Blue, cell lines without KRAS mutations. Orange, cell lines with KRAS mutations. IMR90, non-transformed human lung fibroblasts.

To assess their anti-tumor effects, the compounds in Scheme 1 were tested in a panel of human NSCLC cell lines with or without *KRAS* mutation (Figure 3 and Table 1) [38]. These cell lines are a valuable in vitro platform as they represent the mutational landscape commonly found in NSCLC patients, where loss-of-function mutations of tumor suppressor genes (e.g., TP53, STK11, and CDKN2A) are found in both KRAS mutated and KRAS wild type tumors (Tables S1 and S2 in Supplementary Materials reporting dataset for the cell line mutational status). In general, we found that the Au(I) compounds are more active than those with the oxidized metal centers. In particular, compounds **7** and **8**, with C-Au-P surroundings, exhibited the highest killing potency in MTT assays after 48 h of treatment (Figure 3G,H and Table 1). Au(I) compound **4** with the chemical environment C-Au-Br, with its analog, compound **3**, were particularly effective in those cell lines without a *KRAS* mutation (Figure 3C,D and Table 1). These Au(I) compounds seemed to be particularly effective in those cell lines without KRAS mutation, suggesting that tumors harboring KRAS wild-type might represent a promising candidate for future applications of Au(I)-containing drugs. Since Au(I) compounds are well-known inhibitors of TrxR and inducers of ROS [39] this selectivity might be due to the known intrinsic capability of KRAS to prime anti-oxidant responses during tumorigenesis and tumor progression [40].

The compounds containing the phosphane were more cytotoxic than those containing only the carbene ligands, with the NHC^Me^ ligand being the least effective of those with NHC^Bz^.

The gold(III) compounds were the worst performers, without significant differences between the trichloro or tribromo derivatives (Figure 3A,B,E,F, and Table 1).

The most promising of the compounds, **7** and **8**, were also evaluated in normal non-tumor human lung fibroblasts (IMR90 cell line); both compounds exhibited a good selectivity for the NSCLC cell lines over the lung fibroblasts, suggesting a good therapeutic window in vitro and on-cancer effects (Figure 3G,H and Table 1).

These cytotoxicity results overlap with the stability data, with compounds **7** and **8** being both the most stable in DMSO solution and effective anticancer (Figures S25–S29 in Supplementary Materials).

### 2.3. TrxR Inhibition Activity of the Au Compounds Correlates with Their Cytotoxic Capability

**Figure 4:**
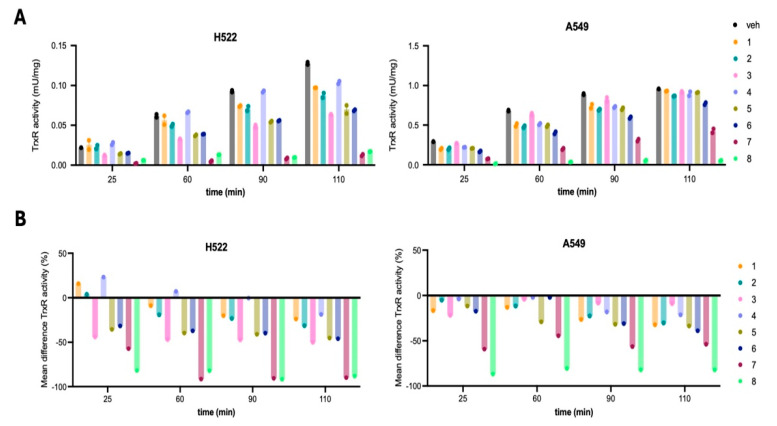
Perturbation of Thioredoxin Reductase activity by the gold compounds in NSCLC cell lines**.** Lysates of H522 and A549 were collected after 24 h of treatment at 5 μM concentration. Assay performed with the Thioredoxin Reductase Assay Kit (Colorimetric) (ab83463). (**A**) Absolute Thioredoxin reductase (TrxR) activity normalized by the total milligrams of protein lysates. (**B**) Mean difference TrxR activity (%) relative to the vehicle (veh) of each time point.

TrxR is a known target of these gold compounds [14,15,16,17,18,19]; therefore, we assayed the inhibitory ability of the different compounds in two representative NSCLC cell lines, H522 (without KRAS mutations) and A549 (with mutant KRAS). The Au(I) compounds **7** and **8**, with C-Au-P surroundings, exhibited the highest degree of TrxR inhibition in both cell lines after 24 h treatment (Figure 4 and Table 2), with compound **8** reaching more than 80% activity reduction on both cell lines.

The NHC-AuX_3_ complexes also consistently inhibited the TxrR activity of about 30–40% (with X = Br, compounds **5** and **6**) and 20–30% (with X = Cl, compounds **1** and **2**) in both cell lines (Figure 4 and Table 2). The lower cytotoxicity and TxrR inhibitory activity of these compounds compared to compounds **7** and **8** might be due to their higher instability in cells and media for a longer period.

**Figure 5:**
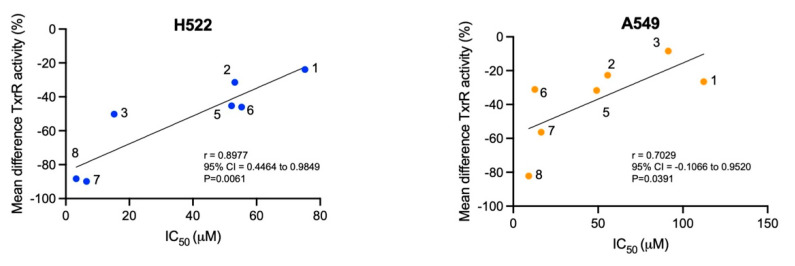
Residual TxrR activity (110 min) correlates with IC_50_ values in lung cancer cell lines**.** Statistics indicate Pearson correlation with alpha = 0.005.

Interestingly, the TxrR inhibitory capability of the gold compounds positively correlates with their killing potency in both H522 and A549 NSCLC cell lines (Figure 5), indicating that TxrR inhibition is involved in the mechanism leading to the cytotoxic effects.

### 2.4. The Trigonal Au(I) Compound **8** with the C-Au-P Environment Inhibits Human DHFR

DHFR is an important target of antiproliferative drugs, being responsible for the maintenance of the tetrahydrofolate intracellular pool needed for one-carbon metabolism; any compounds capable of inhibiting *h*DHFR in fueling one-carbon metabolism with tetrahydrofolate stop cancer cell proliferation. In previous works, we have reported the capability of gold-based drugs to inhibit DHFR from *Escherichia coli* [18], while in the literature, there are no inhibition studies on the human enzyme by this class of compounds.

**Figure 6:**
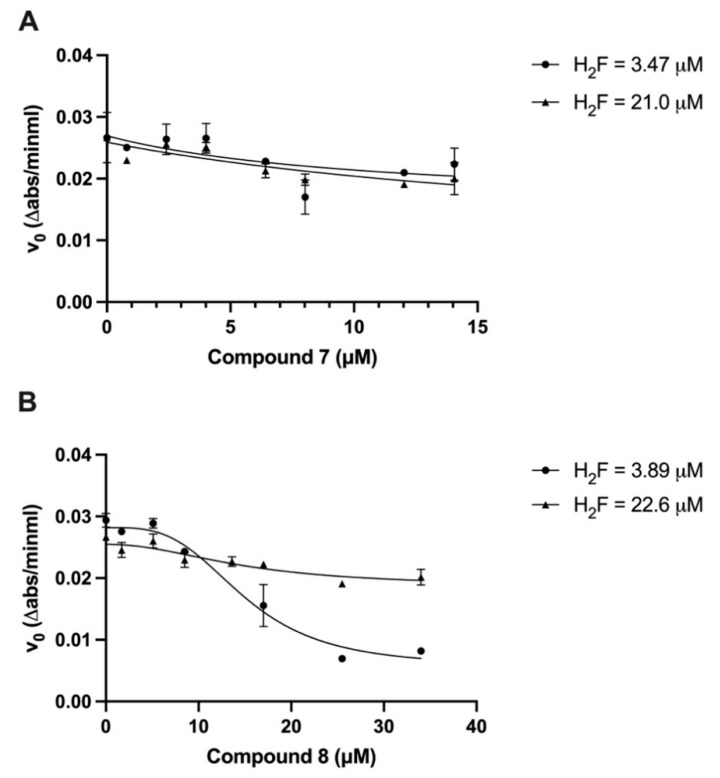
Cell-free *h*DHFR catalyzed reaction velocity is differently inhibited by Au(I) compounds **7** (**A**) and **8** (**B**).

In Figure 6 it is shown that Au(I) compound **7** exhibits very little effect on the enzymatic activity both at sub-saturating (~20 µM) and at close to *Km* (~3 µM) concentrations of the substrate dihydrofolate (H_2_F), while Au(I) compound **8** exerts an important inhibitory function on *h*DHFR inversely dependent on the substrate concentration. Indeed, compound **8** appears to modulate the enzymatic activity of the enzyme through an allosteric mechanism, which is rather unexpected for a monomeric protein. The non-linear sigmoidal fitting model used to interpolate data points in Figure 6B has provided very similar IC_50_ values of 14.97 ± 1.25 and 14.81 ± 1.25 µM at H_2_F concentrations of 3.89 and 22.6 µM respectively. On the other hand, the magnitude of inhibition in terms of residual activity varies very strongly from 80.17 ± 6.97% at an H_2_F concentration of 3.89 µM to 26.96 ± 11.95% at an H_2_F concentration of 22.6 µM. The behavior displayed by the Au(I) compound **7** in affecting the enzymatic activity of *h*DHFR is non-allosteric, and the IC_50_ values are 10.93 ± 9.77 and 23.58 ± 20.74 µM at H_2_F concentrations of 3.47 and 21.0 µM respectively, with a magnitude of inhibition of ~50% at both substrate concentrations. The allosteric inhibition behavior in the presence of the trigonal Au(I) compound **8** could be explained by the presence of a second inhibitor binding site that modifies the structure of the catalytic site, making it less effective in converting the substrate into the tetrahydrofolate product.

## 3. Materials and Methods

### 3.1. Materials

All the reactants needed for the preparation of compounds **1**–**8** were purchased from Merck and used without any further purification. Solvents were bought from Carlo Erba (Milano, Italy) and freshly distilled before use. Celite and molecular sieves were bought from Supelco (Merck). The TrxR assay Kit (ab83463) and the Proteinase Inhibitor Cocktail (ab65621) were purchased from Abcam (Waltham, MA, USA). The plasmid pET17b encoding the cDNA for the *h*DHFR was kindly gifted by prof. C. Robert Matthews (University of Massachusetts Medical School); Hepes buffer, K_2_HPO_4_, NADPH, dihydrofolic acid, and β-mercaptoethanol were purchased from Merck Life Science (Milan, Italy). BL21 competent *E. coli* cells were purchased from Novagen^®^, Merck KGaA (Darmstadt, Germany).

### 3.2. Characterizations

Elemental analyses (C, H, N, S) were performed in-house with a Fisons Instruments 1108 CHNS-O Elemental Analyser. Melting points were taken on an SMP3 Stuart Scientific Instrument. IR spectra were recorded from 4000 to 600 cm^−1^ with a Perkin-Elmer SPECTRUM ONE System FT-IR instrument. IR annotations used: br = broad, m = medium, s = strong, sh = shoulder, vs = very strong, w = weak, and vw = very weak. ^1^H NMR spectra were recorded on an Oxford-400 Varian spectrometer (400.4 MHz for ^1^H). Chemical shifts, in ppm, for ^1^H NMR spectra are relative to internal Me_4_Si. NMR annotations used: br = broad, d = doublet, dd = double doublet, t = triplet, m = multiplet, s = singlet. UV-Vis spectra were acquired using the Shimadzu UV-2700i spectrophotometer, equipped with the Shimadzu CPS-100 Peltier, at 298 K (Shimadzu, Kyoto, Japan). Electrospray mass spectra (ESI-MS) were obtained in positive- or negative-ion mode on a high-performance liquid chromatography (HPLC) Alliance 2695 Waters coupled with a single quadrupole mass spectrometry (Waters Micromass ZQ, Milford, MA, USA). The mobile phase was acetonitrile or methanol, and the compounds were dissolved in the mobile phase with an approximate concentration of 0.1 mM. The injection volume was 1 µL, and the flow rate was 200 µL min^−1^. Nitrogen was employed both as a drying and nebulizing gas. Capillary voltages were typically 4000 V and 3500 V for the positive- and negative-ion modes, respectively. Confirmation of all major species in this ESI-MS study was aided by a comparison of the observed and predicted isotope distribution patterns, the latter calculated using ChemDraw 20.1 computer program.

### 3.3. Preparations

Gold precursors used to obtain the starting NHC^Me,Bz^AuCl compounds were prepared by a classic procedure: HAuCl_4_ was dissolved in ethanol and reacted with an excess of tetrahydrothiophene, dimethylsulfide or PPh_3_ till the precipitation of white THTAuCl, Me_2_SAuCl, or PPh_3_PauCl, respectively [33]. The NHC^Me,Bz^AuCl precursors, 1-benzyl-3-methyl-imidazolyl-2-gold(I)chloride and 1,3-dimethyl-imidazolyl-2-gold(I)chloride, were prepared according to procedures reported in the literature [11,31]. The single crystal X-ray diffraction molecular structures of compounds **1**–**3**, **5**, and **6** are discussed in another article [31]. The molecular structures reported in Scheme 1 are based on these results.

#### 3.3.1. Synthesis of Compound **1**

1,3-dimethyl-imidazol-2yl-gold(I) chloride (100 mg, 0.304 mmol) was dissolved in 10 mL of acetonitrile, then solid iodobenzene dichloride (125.51 mg, 0.457 mmol) was added. The reaction was stirred for 24 h at room temperature under a nitrogen atmosphere. After 24 h, the yellow solution was evaporated under reduced pressure, and the resulting yellow solid was washed with n-hexane (2 × 5 mL) and dried under vacuum, yield: 98%.

^1^H-NMR (DMSO-d^6^, 500 MHz): 7.17 (s, 2 H), 3.19 (s, 3 H).

IR (cm^−1^): ṽ = 3308, 3157, 3122, 2956, 1721, 1697, 1624, 1585, 1574, 1498, 1436, 1411, 1339, 1303, 1279, 1230, 1146, 1084, 1044, 846, 765, 738, 669, 622, 614.

Elemental analysis for C_5_H_8_N_2_AuCl_3_ calcd %: C 15.03; H 2.02; N 7.01; found %: C 14.73; H 2.05; N 6.97.

(+) ESI-MS (CH_3_CN): 131 *m*/*z* (100), 197 *m*/*z* (38) [Au]^+^; 334 *m*/*z* (8) [NHC^Me^Au + CH_3_CN]^+^, 403.5 *m*/*z* (9) [NHC^Me^-AuCl_3_–Cl + CH_3_CN]^+^, at 460.13 *m*/*z* (18) [(NHC^Me^)_2_AuCl_2_]^+^, 758.8 *m*/*z* (16).

#### 3.3.2. Synthesis of Compound **2**

1-benzyl-3-methyl-imidazol-2yl-gold(I) chloride (50 mg, 0.12 mol) was dissolved in 10 mL of acetonitrile, then solid iodobenzene dichloride (50, 9 g, 0.185 mmol) was added. The reaction was stirred for 24 h at room temperature under a nitrogen atmosphere. The yellow solution was evaporated under reduced pressure, and the resulting yellow solid was washed with n-hexane (2 × 5 mL) and dried under vacuum, yield = 88%

^1^H-NMR (DMSO-d^6^, 500 MHz): δ 7.78–7.76 (m, 2 H), 7.43–7.35 (m, 5 H), 5.51 (s, 2 H), 3.91 (s, 3 H).

IR (cm^−1^): ṽ = 3154, 3122, 1568, 1489, 1455, 1406, 1363, 1329, 1240, 1208, 1154, 1080, 786, 760, 732, 706, 696, 680, 645.

Elemental Analysis for C_11_H_12_N_2_AuCl_3_ calcd %: C 27.78; H 2.54; N 5.89; found %: C 27.58; H 2.73; N 6.06.

(+) ESI-MS (CH_3_CN): 173 *m*/*z* (35) [NHC^Bz^]^+^; at 541 *m*/*z* (55) [(NHC^Bz^)_2_Au]^+^; 612 *m*/*z* (100) [(NHC^Bz^)_2_AuCl_2_]^+^.

#### 3.3.3. Synthesis of Compound **3**

1,3-dimethyl-imidazol-2yl-gold(I) chloride (160 mg, 0.487 mmol) was dissolved in 10 mL of acetone, then sodium bromide (420 mg, 4.09 mmol) was added. The reaction was stirred for 24 h at room temperature. The resulting solution was reduced to dryness in a vacuum, redissolved in dichloromethane, and filtered through a bed of celite. The solution was evaporated under reduced pressure, and the resulting white solid was washed with n-hexane (2 × 5 mL) and dried under vacuum, yield = 74%

-NMR (DMSO-d^6^): δ 7.43 (s, 4 H), 3.75 (s, 12 H).

IR (cm^−1^): ṽ = 3149, 3116, 2941, 1563, 1469, 1396, 1347, 1219, 1141, 1076, 749, 726, 655.

Elemental Analysis for C_10_H_16_N_4_Au_2_Br_2_ calcd %: C 16.10; H 2.16; N 7.51; found %: C 16.29; H 2.22; N 7.62.

(+) ESI-MS (CH_3_CN): 111 *m*/*z* (5), 223 *m*/*z* (15), 334 *m*/*z* (8), 389 *m*/*z* (100) [(NHC^Me^)_2_Au]^+^.

(−) ESI-MS (CH_3_CN, *m*/*z*): 356 *m*/*z* (100) [AuBr_2_]^−^

#### 3.3.4. Synthesis of Compound **4**

1-benzyl-3-methyl-imidazol-2yl-gold(I) chloride (125 mg, 0.308 mmol) was dissolved in 10 mL of acetone, then sodium bromide (266, 99 mg, 2.59 mmol) was added. The reaction was stirred for 24 h at room temperature. The resulting solution was reduced to dryness in vacuo, redissolved in dichloromethane, and filtered through a bed of celite. The solution was evaporated under reduced pressure, and the resulting white solid was washed with n-hexane (2 × 5 mL) and dried under vacuum, yield = 91%.

^1^H-NMR (DMSO-d^6^, 500 MHz): δ 7.55 (d, ^3^J_HH_ = 1.9 Hz, 1 H), 7.48 (d, ^3^J_HH_ = 1.9 Hz, 1 H), 7.40–7.33 (m, 5 H), 5.35 (s, 2 H), 3.77 (s, 3 H).

IR (cm^−1^): ṽ = 3155, 3123, 3103, 3028, 2945, 1964, 1819, 1686, 1585, 1565, 1495, 1464, 1451, 1403, 1356, 1306, 1239, 1201, 1190, 1128, 1080, 1045, 1031, 969, 844, 775, 745, 735, 692, 663.

Elemental Analysis for C_11_H_12_N_2_AuBr calcd %: C 29.42; H 2.69; N 6.24; found %: C 29.36; H 2.75; N 6.27.

(+) ESI-MS (CH_3_CN): 173 (15) [NHC^Bz^]^+^; 223 *m*/*z* (13), 465 *m*/*z* (13), 410 (7), 541 *m*/*z* (100) [(NHC^Bz^)_2_Au]^+^.

#### 3.3.5. Synthesis of Compound **5**

[bis-(1,3-dimethyl-imidazoly-2yl)]gold(I) dibromoaurate (50 mg, 0.77 mmol) was dissolved in 1 L of acetonitrile, then liquid bromine (36, 48 mg, 11 µL, 0.227 mmol) were added. The reaction was stirred for 2 h at room temperature under a nitrogen atmosphere. The solution was evaporated under reduced pressure, and the resulting dark orange solid was washed with n-hexane (2 × 5 mL) and dried under vacuum, yield = 93%

-NMR (DMSO-d^6^, 500 MHz): δ 7.73 (s, 2 H), 3.82 (s, 3 H).

IR (cm^−1^): ṽ = 3154, 3121, 2927, 1729, 1700, 1628, 1603, 1572, 1493, 1440, 1411, 1341, 1231, 1086, 754, 740, 686, 666, 615.

Elemental Analysis for C_5_H_8_N_2_AuBr_3_ calcd %: C 11.27; H 1.51; N 5.26; found %: C 11.23; H 1.65; N 5.30.

(+) ESI-MS (CH_3_CN): 111 *m*/*z* (100), 549 *m*/*z* (32) [(NHC^Me^)_2_AuBr_2_]^+^.

#### 3.3.6. Synthesis of Compound **6**

1-benzyl-3-methyl-imidazol-2yl-gold(I) chloride (50 mg, 0.123 mmol) was dissolved in 10 mL of acetonitrile, then liquid bromine (50, 95 mg, 9 µL, 0.185 mmol) was added. The reaction was stirred for 2 h at room temperature under a nitrogen atmosphere. The dark orange solution was then evaporated under reduced pressure, and the resulting solid was washed with n-hexane (2 × 5 mL) and dried under a vacuum, yield = 84%

^1^H-NMR (DMSO-d^6^, 500 MHz): δ 7.77 (d, _HH_ = 2 Hz, 1 H), 7.74 (d, ^3^J_HH_ = 2 Hz, 1 H), 7.42–7.36 (m, 5 H), 5.45 (s, 2 H), 3.84 (s, 3 H).

IR (cm^−1^): ṽ = 3151, 3119, 3027, 2952, 1588, 1567, 1484, 1455, 1403, 1362, 1329, 1239, 1206, 1188, 1158, 1128, 1078, 1030, 1001, 955, 920, 846, 785, 751, 729, 703, 695, 677, 644.

Elemental Analysis for C_11_H_12_N_2_AuBr_3_ calcd %: C 21.70; H 1.99; N 4.60; found %: C 21.70; H 2.10; N 4.62.

(+) ESI-MS (CH_3_CN): 131.8 *m*/*z* (64), 152.4 *m*/*z* (44), 171 *m*/*z* (18), 253 *m*/*z* (100), 270.2 *m*/*z* (99), 288 *m*/*z* (83), 410 *m*/*z* (15), 528.7 (20) [(NHC^Bz^)AuBr_2_]^+^, 569.7 *m*/*z* (72) [(NHC^Bz^)AuBr_2_ + CH_3_CN]^+^, 627.7 *m*/*z* (28), 700.9 *m*/*z* (20), 819 *m*/*z* (10), 978 *m*/*z* (8); 1088 *m*/*z* (31).

#### 3.3.7. Synthesis of Compound **7**

136 mg of the 1-benzyl-4,5-dichloroimidazole (0.6 mmol) were dissolved in 10 mL of anhydrous THF under a nitrogen flux. To this solution, 0.24 mL of n-BuLi (0.6 mmol) was added at −40 °C, and the orange solution was stirred at this temperature for an hour. The solution was heated to 0 °C for 20 min, and 1.5 mL of a 0 °C cooled THF solution of PPh_3_AuPF_6_ (0.6 mmol) was added. The solution was stirred for 1 h and 30 min at 0 °C and 30 min at room temperature. Afterward, the solvent was evaporated, and the brown residue was dissolved in CH_2_Cl_2_ (20 mL) and treated with water (5 × 10 mL). The organic fraction was collected in a flask and dried over Na_2_SO_4_, filtered, and the solution was dried at reduced pressure. The oily residue was then washed overnight with hexane (20 mL). A first crystallization by CHCl_3_/hexane allowed the elimination of solid impurities. The mother liquor afforded 40 mg of the pure product that was collected and dried at reduced pressure.

^1^H-NMR (CDCl_3_, 500 MHz): δ 7.50–7.38 (m, 15H), 7.25–7.21 (m, 5H), 5.32 (s, 2H).

^31^P-NMR (CDCl_3_, 500 MHz): δ 42.09

Elemental analysis for C_28_H_22_N_2_Cl_2_PAu calcd %: C 49.07 H 3.24, N 4.09; found %: C 48.42, H 2.64, N 3.53.

(+) ESI-MS (CH_3_CN): 130.8 *m*/*z* (58), 172 *m*/*z* (47), 212.9 *m*/*z* (38), 296.6 (7), 684.6 (27) [ImCl_2_AuPPh_3_]^+^, 720.8 *m*/*z* [(PPh_3_)_2_Au]^+^, 1142 *m*/*z* (9).

#### 3.3.8. Synthesis of Compound **8**

1,3-dimethylimidazolium gold(I) chloride (50 mg, 0.15 mmol) was dissolved in 10 mL of dry dichloromethane under a nitrogen atmosphere, and triphenylphosphine (33.9 mg, 0.15 mmol) was added. The solution was magnetically stirred for 18 h at room temperature. The solvent was then removed under reduced pressure. The white solid was washed several times with hexane and dried under vacuum overnight, obtaining a white microcrystalline product, yield = 45%.

^1^H-NMR (CDCl_3_, 500 MHz): δ 7.60–7.47 (15 H, m), 7.24 (s, 2 H), 3.98 (s, 6 H).

^1^H-NMR (DMSO-d^6^, 500 MHz): δ 7.67–7.51 (15 H, m), 7.45 (s, 2 H), 3.86 (s, 6 H).

^31^P-NMR (CDCl_3_, 500 MHz): δ 33.22

^31^P-NMR (DMSO-d^6^, 500 MHz): δ 32.45

MIR (cm^−1^): 3348(m), 3117(w), 1652(w), 1567(w), 1479(s), 1433(s), 1361(w), 1312(w), 1238(s), 1177(w), 1101(s), 1026(w), 998(w), 747(vs), 712(s), 690(vs), 679(s), 616(m), 545(vs), 499(vs), 449(m).

Elemental analysis for C_23_H_23_N_2_ClPAu calcd %: C 46.76 H 3.92, N 4.74; found %: C 46.47, H 3.73, N 4.85.

(+) ESI-MS (CH_3_CN): 125 *m*/*z* (8), 195 *m*/*z* (12), 389 (100), 554.9 (22) [NHC^Me^AuPPh_3_]^+^, 585 (9), 720.9 *m*/*z* (10) [(PPh_3_)_2_Au]^+^.

### 3.4. Cell Lines

NSCLC cell lines were obtained from the Hamon Center for Therapeutic Oncology Research (UT Southwestern Medical Center) [38]. IMR-90 human lung fibroblasts were from ATCC (CC-186). All cell lines were maintained and treated in RPMI medium (Cytiva (Shanghai, China), #SH30027), supplemented with 10% heat-inactivated FBS (Millipore Sigma (Darmstadt, Germany), F0394).

### 3.5. MTT Assays

5000–10,000 cells/0.1 mL/well were seeded in 96-well plates to achieve 70% confluence after 24 h. All drug stock solutions were prepared fresh in DMSO at a concentration of 10 mM. The day after cell plating, we added 0.1 mL of medium containing the appropriate drug dilutions. After 48 h of treatment, 10 μL of MTT solution (5 mg/mL) was added to each well. After 4 h incubation, the medium was aspirated, and the formazan salts dissolved in 200 μL DMSO/well for 10 min at 37 °C. The absorbance was read at 570 nm (OD 570 nm). Data are represented as mean ± SD of two independent experiments.

### 3.6. Thioredoxin Reductase (TrxR) Activity

The enzymatic activity of TrxR in the A549 and H522 cell lysates was measured using the TrxR assay Kit (Abcam ab83463) following the manufacturer’s instructions. This kit is based on the TxrR-mediated reduction of the colorless DTNB into TNB2—(yellow). Briefly, cells were plated to reach 70% confluence on a 100-mm dish in one day. They were treated with 5 μM of the gold compounds or the equivalent quantity of DMSO for 24 h. Then, cells were washed in PBS 1X, detached, and lysed in 100 μL of cold TrxR assay buffer supplemented with the Proteinase Inhibitor Cocktail. 20 μg and 40 μg of total proteins were used for the A549 and the H522, respectively. The DTNB substrate was added to samples with or without TrxR inhibitor (provided with the kit) in 96-well plates. The OD412 was measured soon after the substrate addition (A1) and then after 25, 60, 90, and 110 min (An) at 25 °C using a FLUOstar Omega Microplate Reader (BGM Labtech, Chicago, IL, USA). Data analysis was performed following the manufacturer’s instructions. The OD of TNB2, generated by TrxR in every condition, is:

ΔA_412 nm_ = (A_nAB_ − A_nINH_) − (A_B_ − A_1INH_)

where AB is the assay buffer, and INH is the inhibitor. The TrxR activity is:

(2) $$\frac{\Delta B}{\left(Tn-T1\right)\times mg\;of\;protein\;lysate}\times sample\;dilution\;factor=(nmol/min)/mg=mU/mg$$

where ΔB is the TNB amount calculated applying ΔA_412 nm_ to TNB standard curve (in nmol), T1 is the time of the first reading (A_1AB_, and A_1INH_) (in min), Tn is the time of the second reading (A_nAB_ and A_nINH_) (in min). The residual TxrR activity (%) was calculated by normalizing the TxrR activity for each compound with the matching vehicle values at every n time point.

### 3.7. Cell-Free Human Dihydrofolate Reductase (hDHFR) Enzymatic Activity

Recombinant human DHFR was overexpressed and purified from pET17b (+) encoding the *h*DHFR gene/BL21 (Novagene^®^ (Hong Kong, China)) *E. coli* cells as described in the literature [41]. The protein purity was verified as a single band in Coomassie-stained SDS-PAGE. The concentration of the *h*DHFR was measured spectrophotometrically at 280 nm using the extinction coefficient of 28,400 M^−1^ cm^−1^ and resulted to be equal to 14 µM. The activity of the DHFR was followed by recording the absorbance over time at 340 nm in a UV-VIS spectrophotometer Shimadzu UV-2450. The procedure consisted of mixing the 50 mM Hepes buffer (pH 7.3) with saturating concentration NADPH (85–90 mM) and 0.07 mM *h*DHFR, incubating at 37 °C for 5 min, and starting the reaction by adding H_2_F at various concentrations. When testing the inhibition capacity of gold complexes, the reaction mixture was prepared by adding increasing concentrations of the tested compounds before the incubation at 37 °C. Since the gold complexes were solubilized in 100% methanol (HPLC-grade, Carlo Erba), the enzymatic activity of *h*DHFR was also assessed in the presence of the equal volumes of MeOH used in the assays with the gold complexes and eventually subtracted. Data are represented as mean ± SEM of 2 independent experiments and analyzed according to best-fit models using GraphPad Prism (v10).

### 3.8. Statistics, Data Representation, and Data Analysis

Statistical and data analysis was performed using GraphPad Prism (v10). IC_50_ values were calculated using a three-parameter non-linear regression model with log(inhibitor) vs. response. For the TxrR activity statistics, the groups were compared using a two-way ANOVA followed by Dunnett’s multiple comparisons with alpha = 0.05. The correlation between IC_50_ values and TxrR activity was achieved using Pearson correlation with alpha = 0.05.

For the data analysis of *h*DHFR enzymatic activity, the sigmoidal non-linear fitting model in the GraphPad Prism (v10) was used, in which the concentration of inhibitor was expressed as concentrations: Y = Bottom + (Top − Bottom)/(1 + (IC_50_/X)^HillSlope). Figures were partially prepared with BioRender (https://app.biorender.com) accessed on 25 July 2024).

## 4. Conclusions

Among the eight tested gold(I) and gold(III) complexes, Au(I) compounds **7** and **8**, with C-Au-P surroundings, exert the highest cytotoxicity toward a large panel of NSCLC cell lines with or without targetable mutations in oncogenic drivers such as *KRAS*. The NHC-Au complexes without the PPh_3_ moiety, independently of the oxidation state of the central metal, were found to be still active but less effective. A strategy to improve the performance of the NHCAuX and NHC-AuX_3_ complexes could be their encapsulation in nanoparticles, as previously reported for other Au(III) and Au(I) compounds [42,43]. Notably, the most active gold compounds exhibited the highest degree of TrxR inhibition in both H522 and A549 lung cancer cell lines after 24-h treatment, confirming the role of this enzyme on the mechanism of action [11]. As TrxR exerts an important anti-oxidant role, it seems reasonable to think that the Au-compounds would be particularly effective in lung cancer, in which there is a high level of reactive oxygen species (ROS) due to air pollution, inhalable agents, and tobacco smoking.

The anticancer activity of these two promising compounds has also been evaluated as an inhibitor of *h*DHFR, which is one of the most targeted enzymes in chemotherapy drug design. In this context, the Au(I) compounds **7** and **8** have been investigated as potential non-classical, metal-based antifolates. Our results have revealed a most effective inhibition by the trigonal geometry compound **8** over the linear Au compound **7**. Remarkably, compound **8** could reduce the enzymatic activity of *h*DHFR by 80% at a dihydrofolate concentration comparable with the Km, with an IC50 of ~15 mM. This result, even still non-compatible with the classic antifolate nanomolar inhibitory constants, widens the concept of anticancer gold compounds as dual-target inhibitors of the human DHFR and TrxR enzymes [44], making these compounds valuable as alternatives for combination lung anticancer therapies.

Of note, pemetrexed itself, currently used to treat NSCLC patients, inhibits several folate-dependent enzymes such as DHFR, glycinamide ribonucleotide formyl-transferase (GARFT), 5-aminoimidazole-4-carboxamide ribonucleotide formyl-transferase (AICARFT), and thymidylate synthase (TS) which uses 5,10-methylene tetrahydrofolate to convert dUMP into thymidylate, with the release of dihydrofolate in the intracellular folate pool [45].

Studies on lung cancer cell lines have reported the overexpression of thymidylate synthase as one of the mechanisms involved in pemetrexed resistance [46].

The gold complexes **7** and **8** herein studied, by targeting *h*DHFR, in addition to TrxR, can represent alternative drugs to be used in combined therapies, able to resolve the accumulation of dihydrofolate resulting from the TS overexpression in resistant cancer cells. Considering the ability of the gold-based NHC complexes to address a wide array of molecular targets, also observed in other studies [47], they can be exploited as an alternative approach to anticancer drug design, coping with the multiplicity of mechanisms adopted by cancer cells to self-sustain. The structural modularity of complexes **7** and **8** allows the exploration of a broad range of ligand substitutions to optimize the design of new single molecules efficiently targeting both TrxR and DHFR enzymes.

## Figures and Tables

**Table 1:** IC_50_ values of the indicated compounds in human NSCLC cell lines (blue, cell lines without KRAS mutations; orange, cell lines with KRAS mutations). Values indicate mean IC_50_ ± span (μM). Average IC_50_ values across the cell lines of the same group are displayed for each compound; 48 h treatments in MTT assay; N.D. = not determined.

#	H522	H661	H1395	H1993	IC_50_ *	H157	A549	H460	H1792	HCC-44	H1355	IC_50_ *	IMR90
1	75 ± 0.89	47 ± 1.1	47 ± 0.4	37 ± 1.3	52	N.D.	>100	52 ± 1.2	60 ± 1.4	45 ± 0.25	41 ± 0.33	62	N.D.
2	53 ± 2.1	45 ± 1.7	17 ± 0.8	6.1 ± 0.76	30	N.D.	56 ± 1.6	65 ± 2.2	24 ± 0.98	26 ± 1.3	34 ± 1.4	41	N.D.
3	15 ± 1.1	27 ± 1.2	11 ± 0.63	11 ± 0.96	16	N.D.	91 ± 1.03	68 ± 0.10	50 ± 1.2	10 ± 1.1	9.1 ± 1.03	46	N.D.
4	5.3 ± 0.88	7 ± 0.81	11 ± 0.88	12 ± 1.03	9	N.D.	19 ± 1.0	11 ± 1.2	16 ± 0.94	14 ± 1.1	8.1 ± 0.66	14	N.D.
5	52 ± 0.37	42 ± 1.7	49 ± 0.11	70 ± 1.6	53	N.D.	49 ± 0.33	>100	>100	32 ± 1.6	12 ± 0.72	59	N.D.
6	55 ± 1.9	26 ± 1.5	62 ± 2.4	5.6 ± 0.76	37	N.D.	13 ± 1.03	37 ± 1.8	15 ± 1.1	19 ± 1.4	59 ± 1.7	29	N.D.
7	6.5 ± 0.97	0.95 ± 1.0	6.3 ± 0.98	1.8 ± 0.96	4	8.5 ± 1.2	16 ± 1.3	6.6 ± 1.02	12 ± 1.04	9.2 ± 1.02	7.8 ± 0.61	10	63 ± 2.5
8	3.3 ± 0.93	1.1 ± 1.1	5.9 ± 0.97	1.8 ± 1.05	3	17 ± 1.2	9.1 ± 1.2	13 ± 1.4	15 ± 1.1	7.3 ± 1.05	6.4 ± 0.65	11	87 ± 4.9

* Averaged values.

**Table 2:** Variation and statistics of the TxrR activity in H522 and A549 lung cancer cell lysates collected after 24 h of treatment (5 μM). The reduction of DTNB into TNB (OD_412_) was measured at 25, 60, 90, and 110 min. Two-way ANOVA followed by Dunnett’s multiple comparisons with ns = *p* > 0.05; * = *p* > 0.05; ** = *p* > 0.01; *** = *p* > 0.001 and **** = *p* > 0.0001.

Dunnett’s Comparison	H522				A549			
	Mean Diff.	95.00% CI of Diff.	Summary	Adj *p* Value	Mean Diff.	95.00% CI of Diff.	Summary	Adj *p* Value
25 min								
1 vs. veh	15.84	−6.504 to 38.19	ns	0.2655	−16.73	−32.31 to −1.152	*	0.0306
2 vs. veh	3.864	−18.48 to 26.21	ns	0.9981	−5.148	−20.73 to 10.43	ns	0.918
3 vs. veh	−44.05	−66.40 to −21.70	****	<0.0001	−21.88	−37.46 to −6.300	**	0.0027
4 vs. veh	23.18	0.8373 to 45.53	*	0.0391	−3.861	−19.44 to 11.72	ns	0.9819
5 vs. veh	−35.55	−57.90 to −13.20	***	0.0006	−11.58	−27.16 to 3.996	ns	0.2233
6 vs. veh	−31.68	−54.03 to −9.338	**	0.0024	−17.37	−32.95 to −1.795	*	0.023
7 vs. veh	−57.19	−79.53 to −34.84	****	<0.0001	−59.2	−74.78 to −43.62	****	<0.0001
8 vs. veh	−81.92	−104.3 to −59.57	****	<0.0001	−86.87	−102.5 to −71.29	****	<0.0001
60 min								
1 vs. veh	−8.894	−31.24 to 13.45	ns	0.8206	−13.1	−28.68 to 2.479	ns	0.1319
2 vs. veh	−19.16	−41.50 to 3.190	ns	0.1201	−11.64	−27.22 to 3.934	ns	0.2189
3 vs. veh	−47.07	−69.42 to −24.72	****	<0.0001	−4.221	−19.80 to 11.36	ns	0.97
4 vs. veh	7.116	−15.23 to 29.46	ns	0.9312	−2.183	−17.76 to 13.40	ns	0.9996
5 vs. veh	−39.68	−62.03 to −17.34	***	0.0001	−29.11	−44.69 to −13.53	****	<0.0001
6 vs. veh	−37.22	−59.57 to −14.87	***	0.0003	−2.183	−17.76 to 13.40	ns	0.9996
7 vs. veh	−91.41	−113.8 to −69.06	****	<0.0001	−44.4	−59.97 to −28.82	****	<0.0001
8 vs. veh	−82.1	−104.4 to −59.75	****	<0.0001	−80.79	−96.36 to −65.21	****	<0.0001
90 min								
1 vs. veh	−20.1	−42.44 to 2.252	ns	0.0938	−26.55	−42.13 to −10.98	***	0.0002
2 vs. veh	−23.38	−45.73 to −1.037	*	0.0368	−22.67	−38.25 to −7.090	**	0.0018
3 vs. veh	−47.31	−69.66 to −24.97	****	<0.0001	−8.42	−24.00 to 7.159	ns	0.5454
4 vs. veh	−0.3654	−22.71 to 21.98	ns	>0.9999	−18.13	−33.71 to −2.556	*	0.0163
5 vs. veh	−41.1	−63.45 to −18.76	****	<0.0001	−31.74	−47.31 to −16.16	****	<0.0001
6 vs. veh	−39.82	−62.17 to −17.48	***	0.0001	−31.09	−46.67 to −15.51	****	<0.0001
7 vs. veh	−90.61	−113.0 to −68.26	****	<0.0001	−56.35	−71.93 to −40.77	****	<0.0001
8 vs. veh	−91.89	−114.2 to −69.54	****	<0.0001	−82.25	−97.83 to −66.68	****	<0.0001
110 min								
1 vs. veh	−23.89	−46.24 to −1.545	*	0.0316	−32.38	−47.96 to −16.80	****	<0.0001
2 vs. veh	−31.46	−53.80 to −9.111	**	0.0026	−30.44	−46.02 to −14.86	****	<0.0001
3 vs. veh	−50.17	−72.52 to −27.83	****	<0.0001	−9.068	−24.65 to 6.512	ns	0.4662
4 vs. veh	−18.72	−41.06 to 3.631	ns	0.1345	−21.37	−36.95 to −5.794	**	0.0034
5 vs. veh	−45.26	−67.61 to −22.91	****	<0.0001	−33.68	−49.26 to −18.10	****	<0.0001
6 vs. veh	−46.06	−68.40 to −23.71	****	<0.0001	−38.86	−54.44 to −23.28	****	<0.0001
7 vs. veh	−89.86	−112.2 to −67.51	****	<0.0001	−53.76	−69.34 to −38.18	****	<0.0001
8 vs. veh	−88.27	−110.6 to −65.92	****	<0.0001	−82.25	−97.83 to −66.68	****	<0.0001

## Data Availability

Data is contained within the article and Supplementary Materials.

## Supplementary Materials

The following supporting information can be downloaded at: https://www.mdpi.com/article/10.3390/ph17091133/s1, IR spectra (Figure S1), ^1^H NMR spectra (Figures S2–S9), ESI-MS spectra (Figures S10–S18), and UV-visible spectra in DMSO or CH_3_OH of compounds **1**–**8** (Figures S19–S29). Cell line mutational status can be found in Supplementary Materials as Tables S1 and S2.

## References

[1] Siegel R.L., Miller K.D., Wagle N.S., Jemal A. (2023). Cancer statistics, 2023. CA Cancer J. Clin..

[2] Shao W., Mishina Y.M., Feng Y., Caponigro G., Cooke V.G., Rivera S., Wang Y., Shen F., Korn J.M., Griner L.M.A. (2018). Antitumor Properties of RAF709, a Highly Selective and Potent Inhibitor of RAF Kinase Dimers, in Tumors Driven by Mutant RAS or BRAF. Cancer Res..

[3] Leiter A., Veluswamy R.R., Wisnivesky J.P. (2023). The global burden of lung cancer: Current status and future trends. Nat. Rev. Clin. Oncol..

[4] Hirsch F.R., Scagliotti G.V., Mulshine J.L., Kwon R., Curran W.J., Wu Y.-L., Paz-Ares L. (2017). Lung cancer: Current therapies and new targeted treatments. Lancet.

[5] Corcoran R.B. (2023). A single inhibitor for all KRAS mutations. Nat. Cancer.

[6] Luo J., Ostrem J., Pellini B., Imbody D., Stern Y., Solanki H.S., Haura E.B., Villaruz L.C. (2022). Overcoming KRAS-Mutant Lung Cancer.

[7] Mármol I., Quero J., Rodríguez-Yoldi M.J., Cerrada E. (2019). Gold as a Possible Alternative to Platinum-Based Chemotherapy for Colon Cancer Treatment. Cancers.

[8] Lum C.T., Sun R.W.-Y., Zoua T., Che C.-M. (2014). Gold(III) complexes inhibit growth of cisplatin-resistant ovarian cancer in association with upregulation of proapoptotic PMS2 gene. Chem. Sci..

[9] Juzheng Z., Yanping L., Ronghao F., Wei W., Yong W., Jiamin J., Feng Y., Jian C. (2022). Organometallic gold(I) and gold(III) complexes for lung cancer treatment. Front. Pharmacol..

[10] Arambula J.F., McCall R., Sidoran K.J., Magda D., Mitchell N.A., Bielawski C.W., Lynch V.M., Sesslerg J.L., Arumug K. (2016). Targeting antioxidant pathways with ferrocenylated N-heterocyclic carbene supported gold(i) complexes in A549 lung cancer cells. Chem. Sci..

[11] Galassi R., Burini A., Ricci S., Pellei M., Rigobello M.P., Citta A., Dolmella A., Gandin V., Marzano C. (2012). Synthesis and characterization of azolate gold(I) phosphane complexes as thioredoxin reductase inhibiting antitumor agents. Dalton Trans..

[12] Gambini V., Tilio M., Maina E.W., Andreani C., Bartolacci C., Wang J., Iezzi M., Ferraro S., Ramadori A.T., Simon O.C. (2018). In vitro and in vivo studies of gold(I) azolate/phosphane complexes for the treatment of basal like breast cancer. Eur. J. Med. Chem..

[13] Martynova E.A., Scattolin T., Cavarzerani E., Peng M., Van Hecke K., Rizzolio F., Nolan S.P. (2022). A simple synthetic entryway into new families of NHC–gold-amido complexes and their in vitro antitumor activity. Dalton Trans..

[14] Ott I. (2009). On the medicinal chemistry of gold complexes as anticancer drugs. Coord. Chem. Rev..

[15] Galassi R., Luciani L., Gambini V., Vincenzetti S., Lupidi G., Amici A., Marchini C., Wang J., Pucciarelli S. (2020). Multi-Targeted Anticancer Activity of Imidazolate Phosphane Gold(I) Compounds by Inhibition of DHFR and TrxR in Breast Cancer Cells. Front. Chem..

[16] Casagrande N., Borghese C., Corona G., Aldinucci D., Adam A., Sulaiman A.A., Isab A.A., Ahmad S., Peedikakkal A.M.P. (2023). Dinuclear gold(I) complexes based on carbene and diphosphane ligands: Bis [2-(dicyclohexylphosphano)ethyl]amine complex inhibits the proteasome activity, decreases stem cell markers and spheroid viability in lung cancer cells. JBIC J. Biol. Inorg. Chem..

[17] Fan C., Zheng W., Fu X., Li X., Wong Y.-S., Chen T. (2014). Enhancement of auranofin-induced lung cancer cell apoptosis by selenocystine, a natural inhibitor of TrxR1 in vitro and in vivo. Cell Death Dis..

[18] Bindoli A., Rigobello M.P., Scutari G., Gabbiani C., Casini A., Messori L. (2009). Thioredoxin reductase: A target for gold compounds acting as potential anticancer drugs. Coord. Chem. Rev..

[19] Nobili S., Mini E., Landini I., Gabbiani C., Casini A., Messori L. (2010). Gold compounds as anticancer agents: Chemistry, cellular pharmacology, and preclinical studies. Med. Res. Rev..

[20] Alhoshani A., Alrashdi A., Alhosaini K., Alanazi F.E., Alajez N.M., Altaf M., Isab A.A., Korashy H.M. (2018). Gold-containing compound BDG-I inhibits the growth of A549 lung cancer cells through the deregulation of miRNA expression. Saudi Pharm. J..

[21] Casini A., Hartinger C., Gabbiani C., Mini E., Dyson P.J., Keppler B.K., Messori L. (2008). Gold(III) compounds as anticancer agents: Relevance of gold-protein interactions for their mechanism of action. J. Inorg. Biochem..

[22] Zoppi C., Messori L., Pratesi A. (2020). ESI MS studies highlight the selective interaction of Auranofin with protein free thiols. Dalton Trans..

[23] Pucciarelli S., Vincenzetti S., Ricciutelli M., Simon O.C., Ramadori A.T., Luciani L., Galassi R. (2019). Studies on the Interaction between Poly-Phosphane Gold(I) Complexes and Dihydrofolate Reductase: An Interplay with Nicotinamide Adenine Dinucleotide Cofactor. Int. J. Mol. Sci..

[24] Liu W., Bensdorf K., Proetto M., Abram U., Hagenbach A., Gust G. (2011). NHC Gold Halide Complexes Derived from 4,5-Diarylimidazoles: Synthesis, Structural Analysis, and Pharmacological Investigations as Potential Antitumor Agents. J. Med. Chem..

[25] Liu C.T., Hanoian P., French J.B., Pringle T.H., Hammes-Schiffer S., Benkovic S.J. (2013). Functional significance of evolving protein sequence in dihydrofolate reductase from bacteria to humans. Proc. Natl. Acad. Sci. USA.

[26] Hamed K.M., Dighriri I.M., Baomar A.F., Alharthy B.T., Alenazi F.E., Alali G.H., Alenazy R.H., Alhumaidi N.T., Alhulayfi D.H., Alotaibi Y.B. (2022). Overview of Methotrexate Toxicity: A Comprehensive Literature Review. Cureus.

[27] Gridelli C., Maione P., Rossi A., Bareschino M.A., Schettino C., Sacco P.C., Zeppa R. (2011). Pemetrexed in advanced non-small cell lung cancer. Expert Opin. Drug Saf..

[28] Chan M., Gravel M., Bramoullé A., Bridon G., Avizonis D., Shore G.C., Roulston A. (2014). Synergy between the NAMPT inhibitor GMX1777(8) and pemetrexed in non-small cell lung cancer cells is mediated by PARP activation and enhanced NAD consumption. Cancer Res..

[29] Hussein E.M., Alsantali R.I., Abd El-Galil S.M., Obaid R.J., Alharbi A., Abourehab M.A.S., Ahmed S.A. (2019). Bioactive fluorenes. part I. Synthesis, pharmacological study and molecular docking of novel dihydrofolate reductase inhibitors based-2,7-dichlorofluorene. Heliyon.

[30] Vásquez A.F., Gómez L.A., González Barrios A., Riaño-Pachón D.M. (2022). Identification of Active Compounds against Melanoma Growth by Virtual Screening for Non--Classical Human DHFR Inhibitors. Int. J. Mol. Sci..

[31] Sargentoni N., Galassi R., Luciani L., Rominger F., Rudolph M., Hashmi A.S.K. (2024). Oxidation state and halogen influence on the NHC-gold-halide catalyzed cyclization of propargyl amides. Adv. Synth. Catal..

[32] Galassi R., Sargentoni N., Luciani L., Manca G., Ienco A. (2024). Halogen addition to NHC-gold(I) chloride complexes in the framework of the Inverted Ligand Field. Inorg. Chim. Acta.

[33] Luciani L., Sargentoni N., Graiff C., Monge M., Rodríguez-Castillo M., López-de-Luzuriaga J.M., Galassi R. (2023). Mechanochemical preparation of strongly emissive monosubstituted triarylphosphane gold(I) compounds activated by hydrogen bonding driven aggregations. RSC Adv..

[34] Hirtenlehner C., Krims C., Hölbling J., List M., Zabel M., Fleck M., Berger R.J.F., Schoefberger W., Monkowius U. (2011). Syntheses, crystal structures, reactivity, and photochemistry of gold(III) bromides bearing N-heterocyclic carbenes. Dalton Trans..

[35] Crabtree R.H. (2005). NHC ligands versus cyclopentadienyls and phosphines as spectator ligands in organometallic catalysis. J. Organomet. Chem..

[36] Huynh H.V., Guo S., Wu W. (2013). Detailed Structural, Spectroscopic, and Electrochemical Trends of Halido Mono- and Bis(NHC) Complexes of Au(I) and Au(III). Organometallics.

[37] de Frémont P., Singh R., Stevens E.D., Petersen J.L., Nolan S.P. (2007). Synthesis, Characterization and Reactivity of N-Heterocyclic Carbene Gold(III) Complexes. Organometallics.

[38] Gazdar A.F., Girard L., Lockwood W.W., Lam W.L., Minna J.D. (2010). Lung Cancer Cell Lines as Tools for Biomedical Discovery and Research. JNCI J. Natl. Cancer Inst..

[39] Leiya K., Shuang W., Pei K. (2021). Current Progress and Perspectives on Using Gold Compounds for the Modulation of Tumor Cell Metabolism. Front. Chem..

[40] Lim J.K.M., Leprivier G. (2019). The impact of oncogenic RAS on redox balance and implications for cancer development. Cell Death Dis..

[41] Wallace L.A., Matthews C.R. (2002). Highly divergent dihydrofolate reductases conserve complex folding mechanism. J. Mol. Biol..

[42] Moreno-Alcántar G., Picchetti P., Casini A. (2023). Gold Complexes in Anticancer Therapy: From New Design Principles to Particle-Based Delivery Systems. Angew. Chem. Int. Ed..

[43] Astolfi P., Pisani M., Giorgini E., Rossi B., Damin A., Vita F., Francescangeli O., Luciani L., Galassi R. (2020). Synchrotron Characterization of Hexagonal and Cubic Lipidic Phases Loaded with Azolate/Phosphane Gold(I) Compounds: A New Approach to the Uploading of Gold(I)-Based Drugs. Nanomaterials.

[44] Ng H.L., Ma X., Chew E.H., Chui W.K. (2017). Design, Synthesis, and Biological Evaluation of Coupled Bioactive Scaffolds as Potential Anticancer Agents for Dual Targeting of Dihydrofolate Reductase and Thioredoxin Reductase. J. Med. Chem..

[45] Liang J., Lu T., Chen Z., Zhan C., Wang Q. (2019). Mechanisms of resistance to pemetrexed in non-small cell lung cancer. Transl. Lung Cancer Res..

[46] Ozasa H., Oguri T., Uemura T., Miyazaki M., Maeno K., Sato S., Ueda R. (2010). Significance of thymidylate synthase for resistance to pemetrexed in lung cancer. Cancer Sci..

[47] Bär S.I., Gold M., Schleser S.W., Rehm T., Bär A., Köhler L., Carnell L.R., Biersack B., Schobert R. (2021). Guided Antitumoural Drugs: (Imidazol-2-ylidene)(L)gold(I) Complexes Seeking Cellular Targets Controlled by the Nature of Ligand, L. Chemistry.

