# Supplementary material for: Anticancer Activity of Imidazolyl Gold(I/III) Compounds in Non-Small Cell Lung Cancer Cell Lines
Source: Pharmaceuticals (Basel). 2024 Aug 28;17(9):1133. doi: 10.3390/ph17091133 (PMC11435220; doi:10.3390/ph17091133)
Supplement: Supplementary file 1 [file pharmaceuticals-17-01133-s001.zip › pharmaceuticals-3101055-supplementary.pdf]

IR spectroscopy

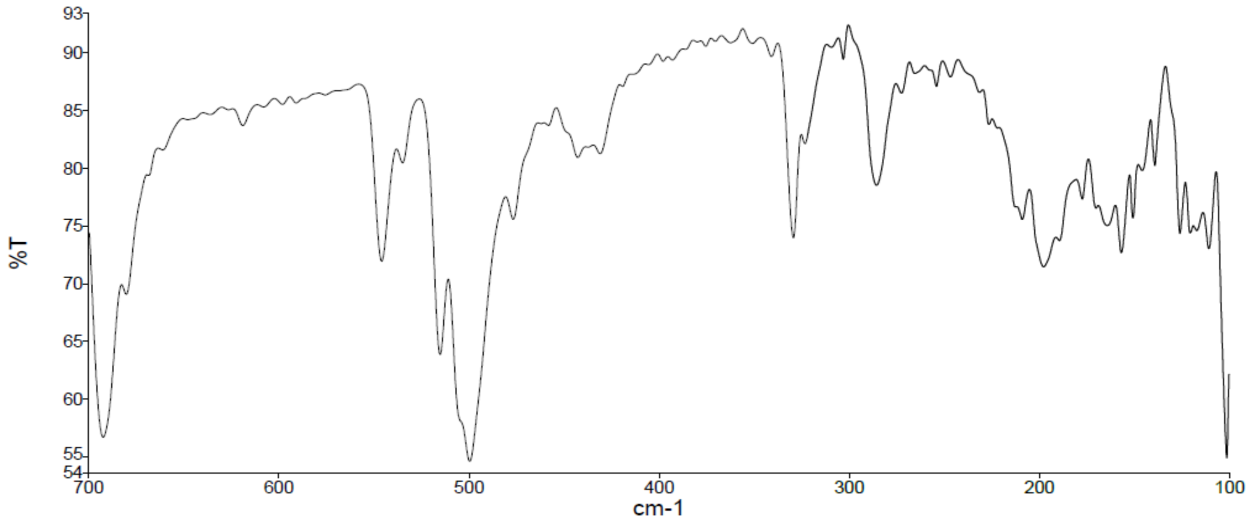

Figure S1. FIR spectrum of compound 8.

¹H NMR spectra

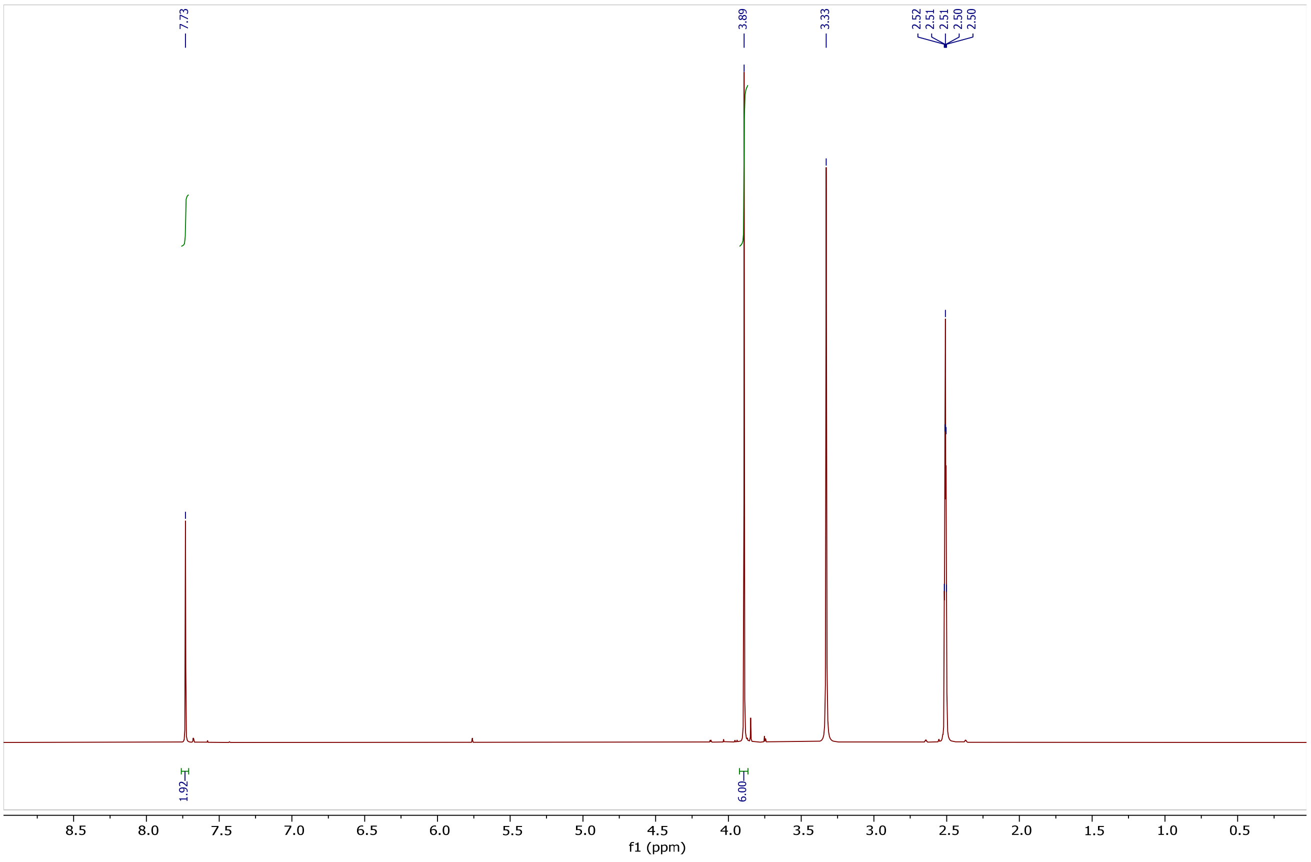

Figure S2. ¹H NMR of complex **1** in DMSO.

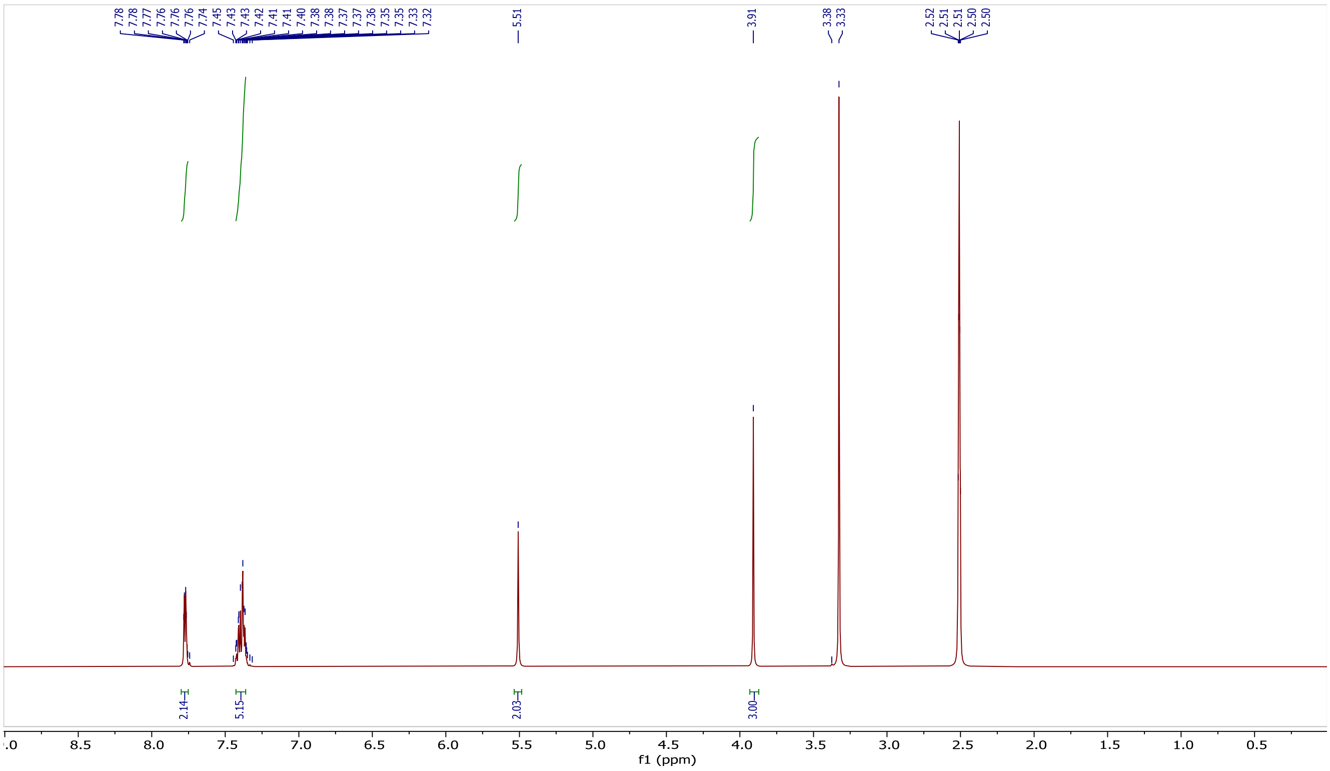

Figure S3. ^1H NMR of complex **2** in DMSO.

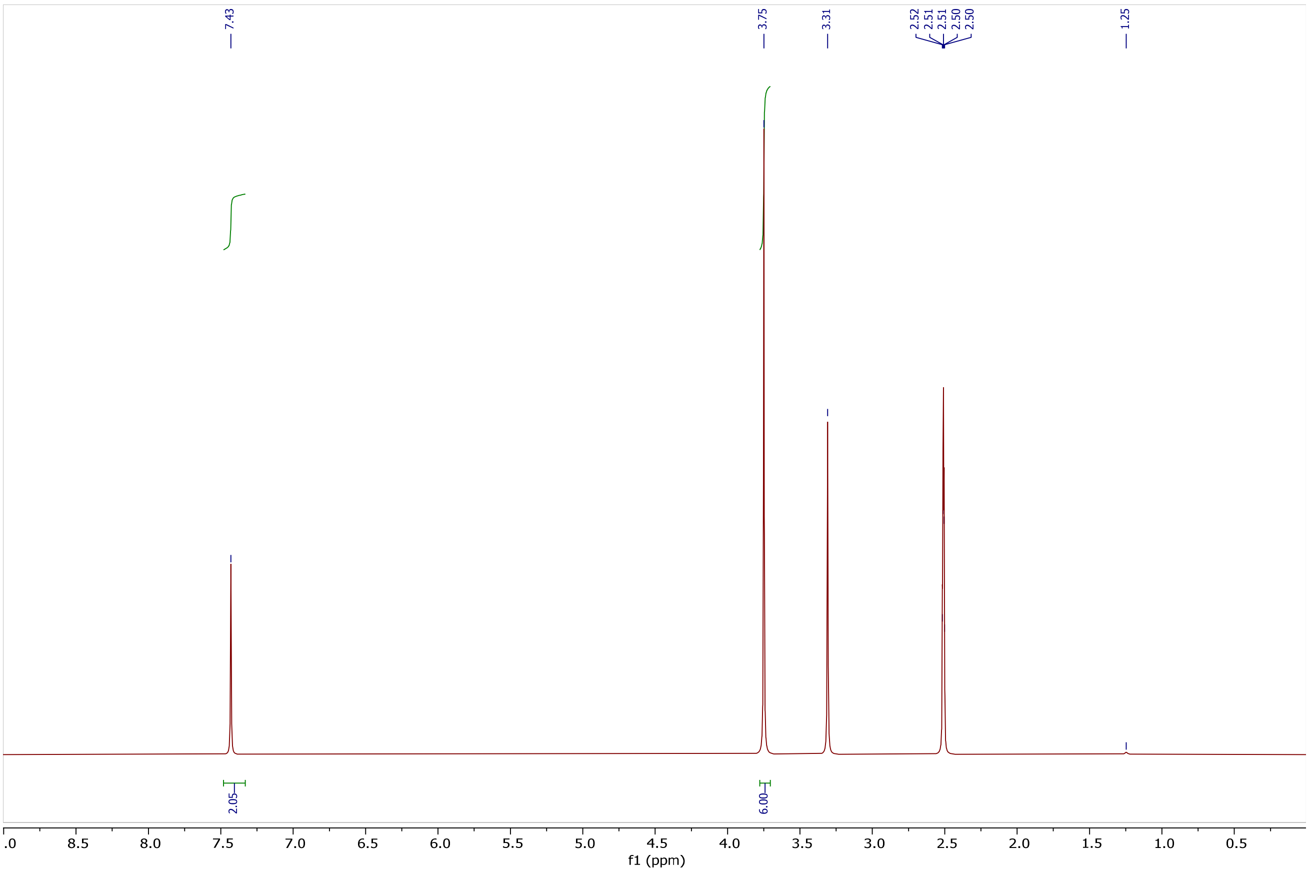

Figure S4. ^1H NMR of complex **3** in DMSO.

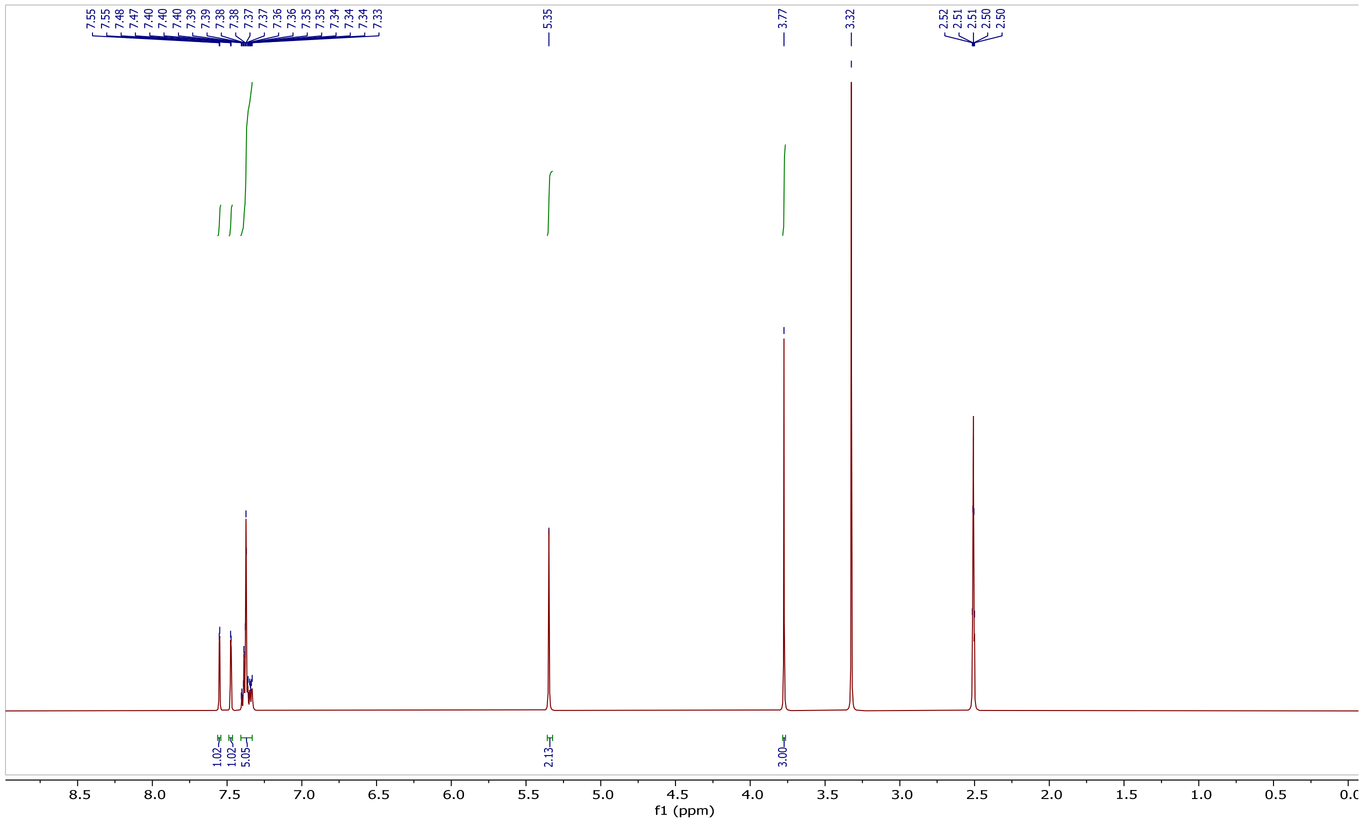

Figure S5. ^1H NMR of complex **4** in DMSO.

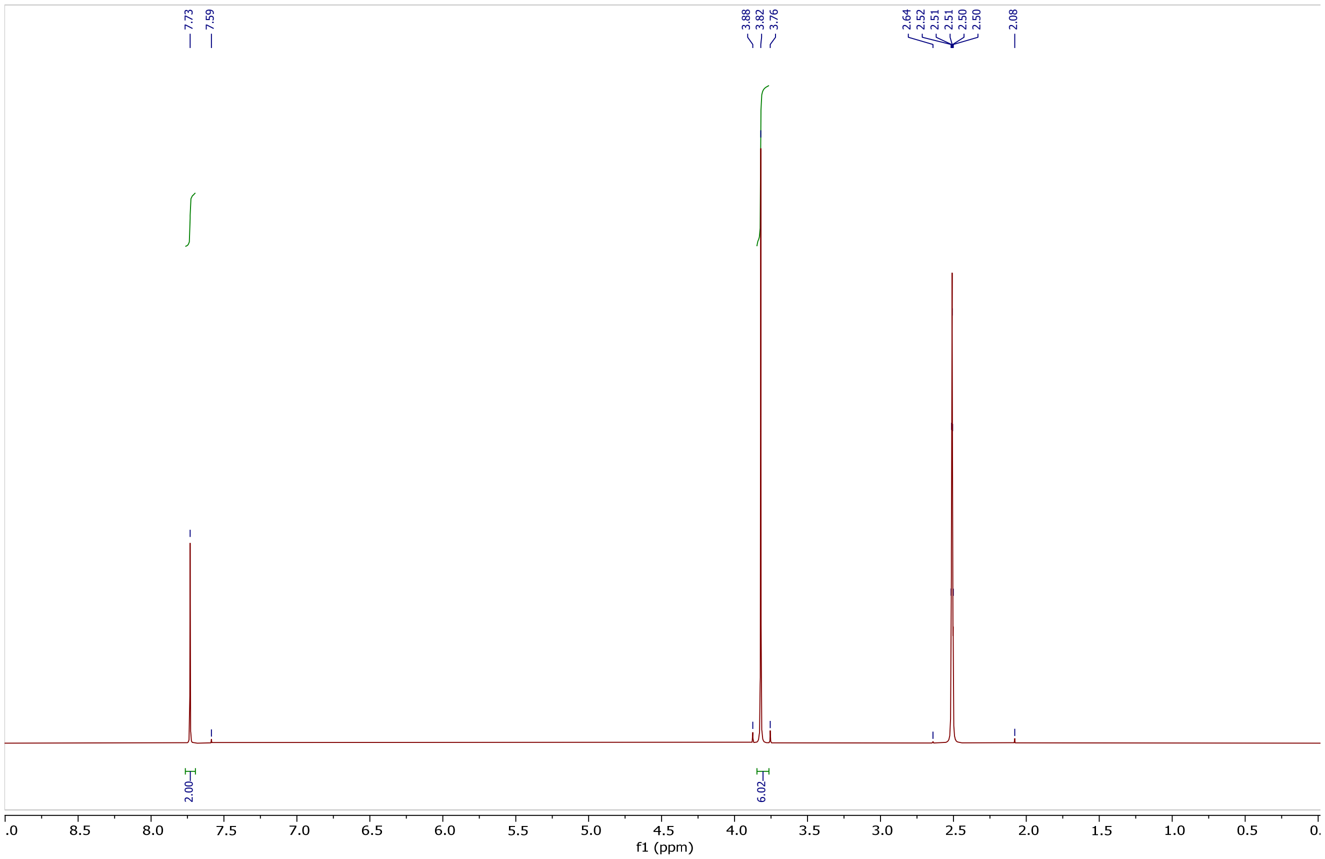

Figure S6. ^1H NMR of complex **5** in DMSO.

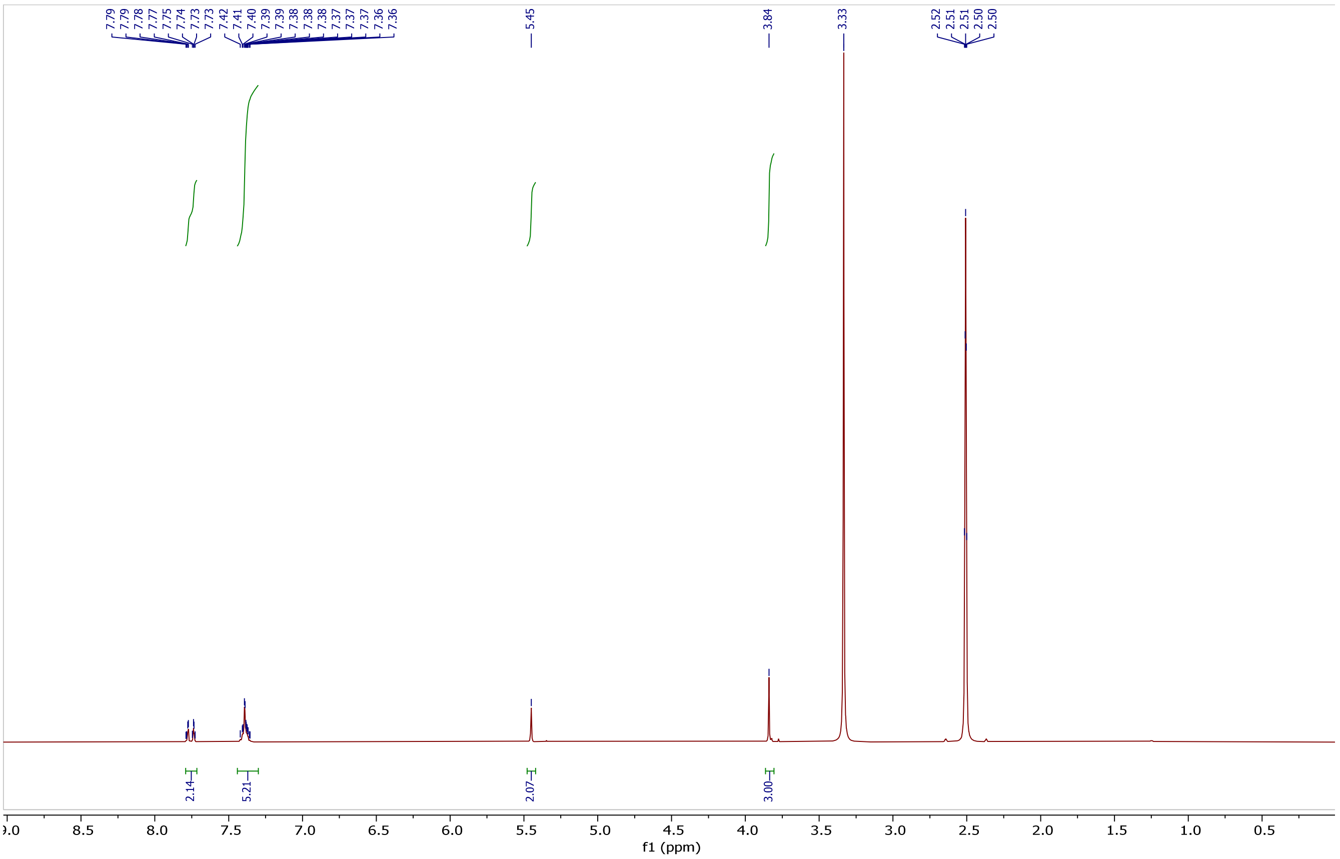

Figure S7. ^{1}H NMR of complex **6** in DMSO.

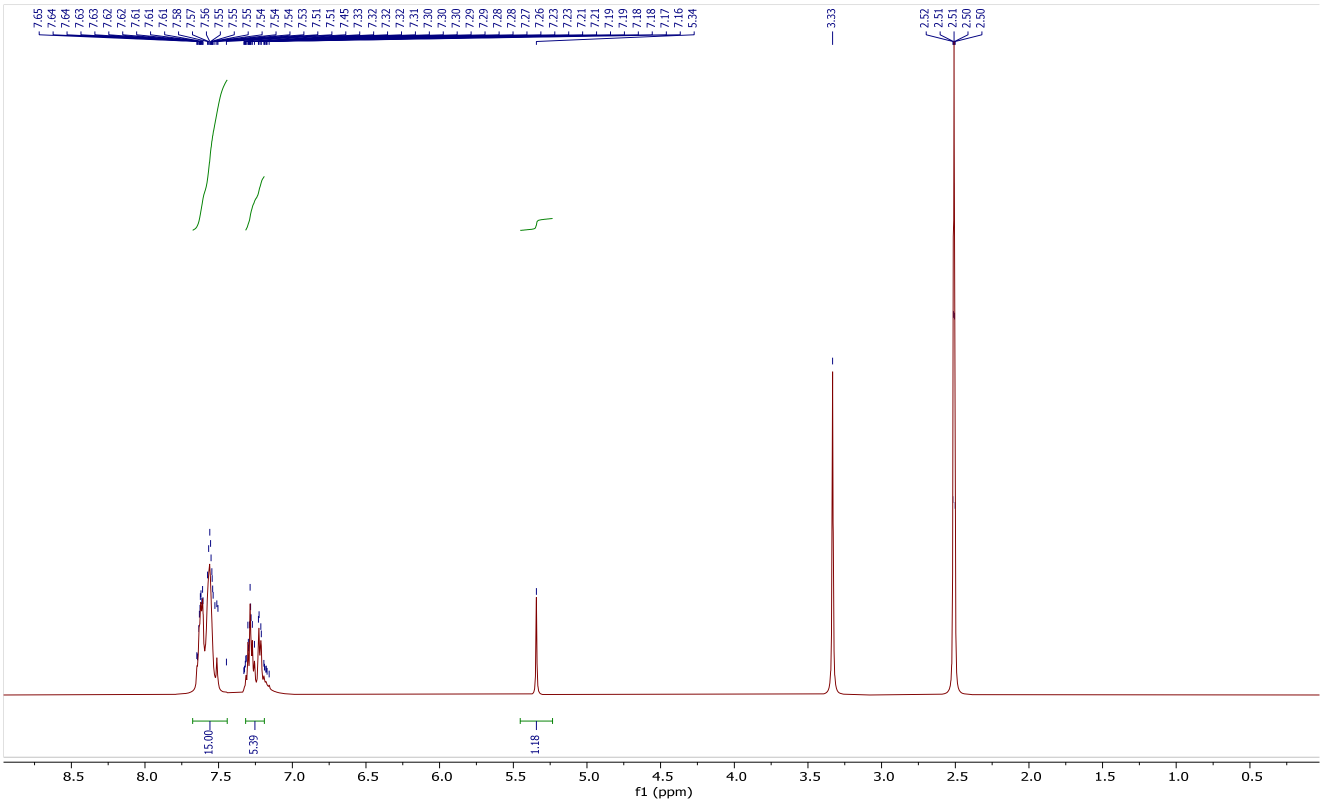

Figure S8. ^{1}H NMR of complex **7** in DMSO.

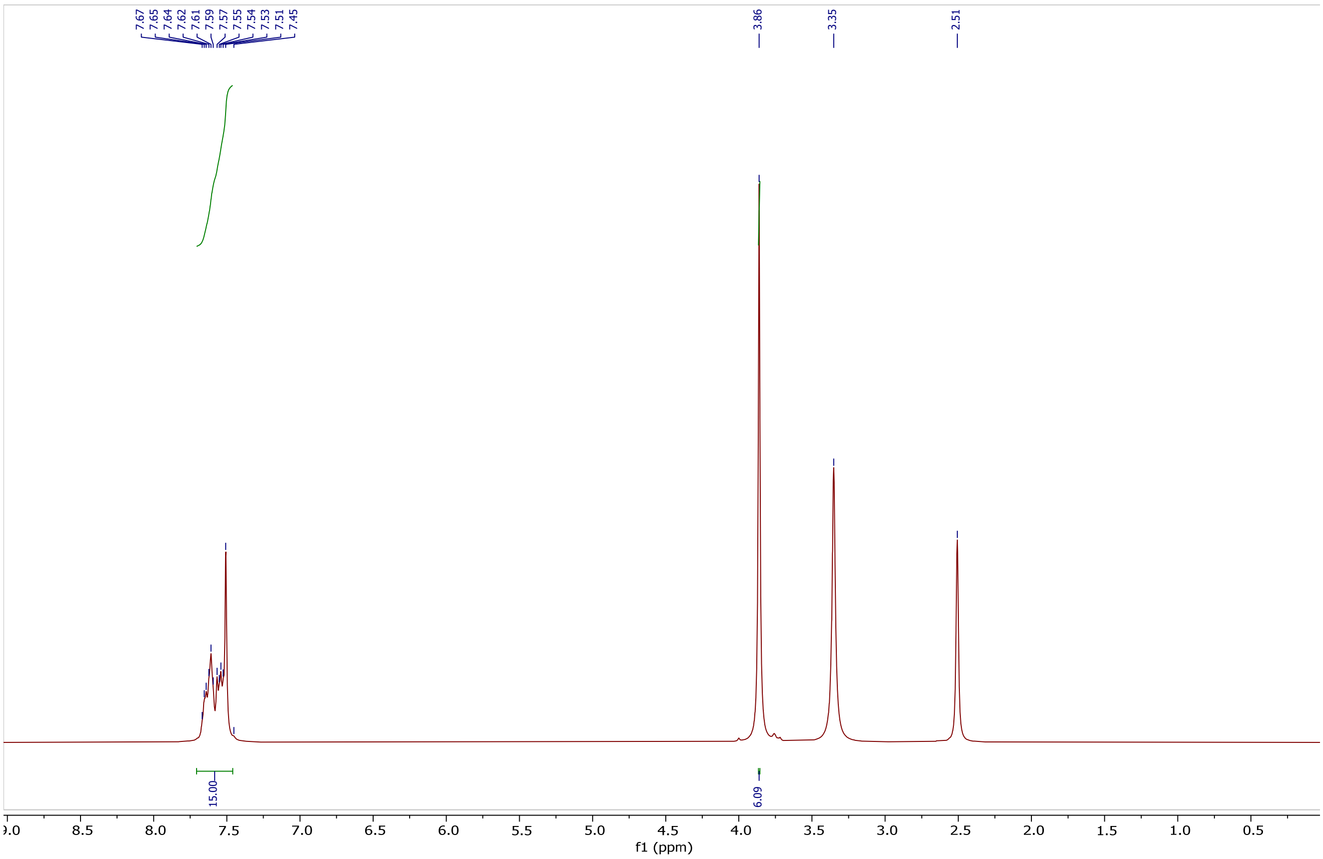

Figure S9. ^{1}H NMR of complex **8** in DMSO.

ESI MS spectra

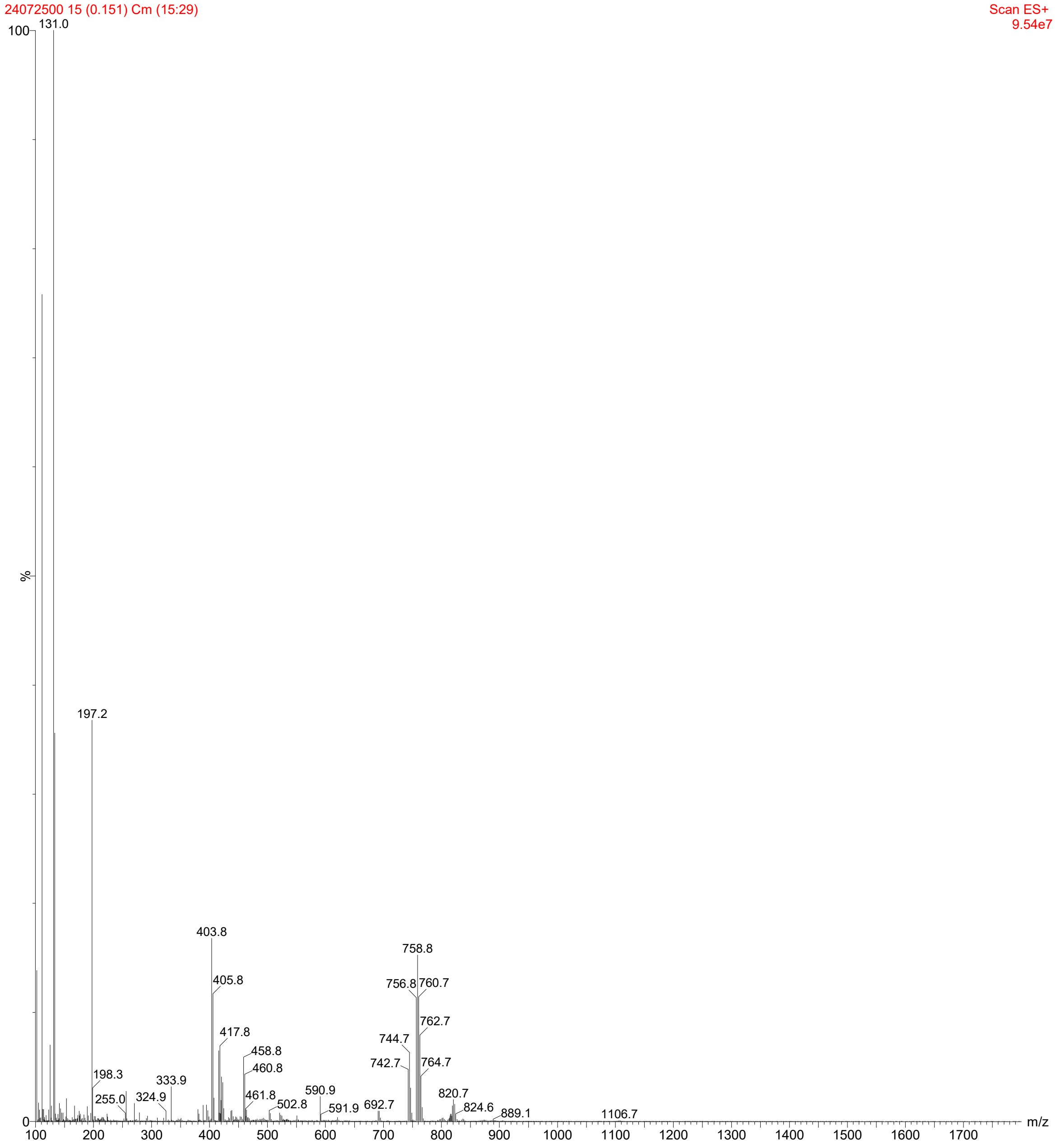

Figure S10. (+) ESI-MS spectrum for compound **1**. Relevant fragments at 334 m/z attributable to $[NHC^{Me}Au + CH_3CN]^+$, at 403.5 m/z attributable to $[NHC^{Me}\text{-}AuCl_3 - Cl + CH_3CN]^+$, at 460.13 m/z attributable to $[(NHC^{Me})_2AuCl_2]^+$.

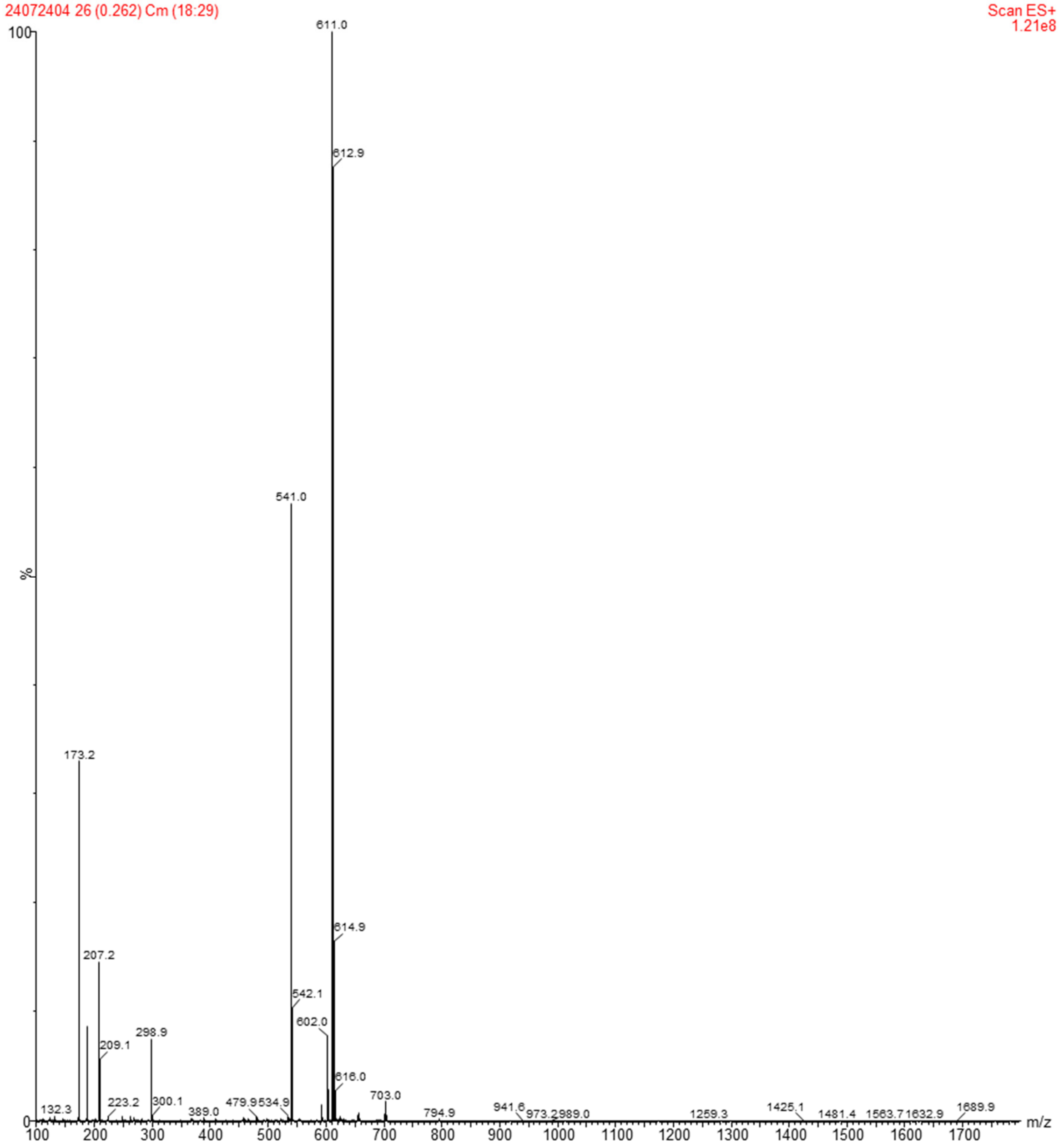

Figure S11. (+)ESI-MS spectrum for compound **2**. Relevant fragment at 541 m/z attributable to $[(NHC^{Bz})_2Au]^+$; 612 m/z attributable to $[(NHC^{Bz})_2AuCl_2]^+$.

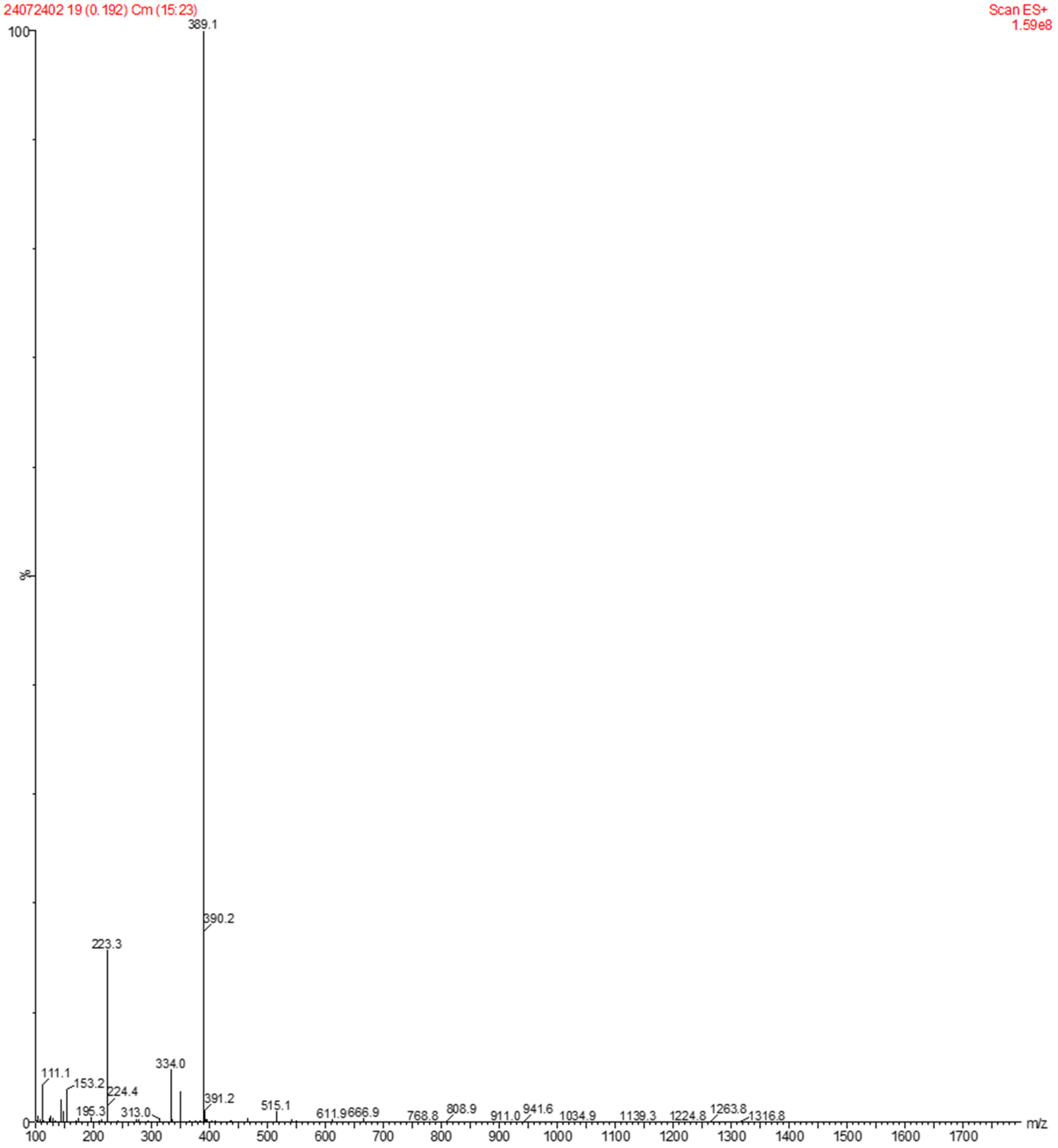

Figure S12. (+)ESI-MS spectrum for compound **3**. Relevant fragments at 389 m/z attributable to $[(NHC^{Me})_2Au]^+$.

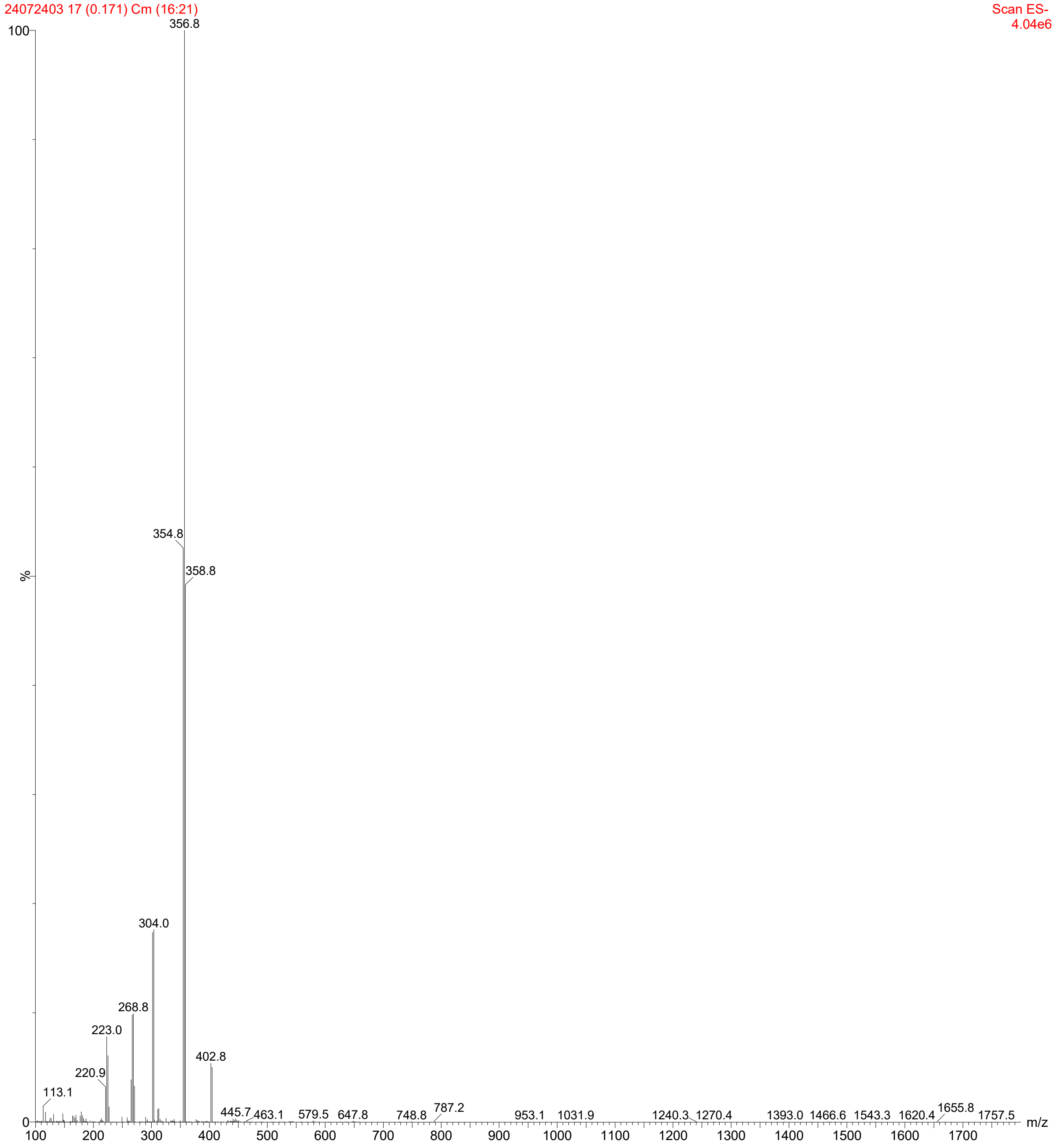

Figure S13. (-)ESI MS for compound **3**. Relevant fragment at 356.8 m/z attributable at $[AuBr_2]^-$.

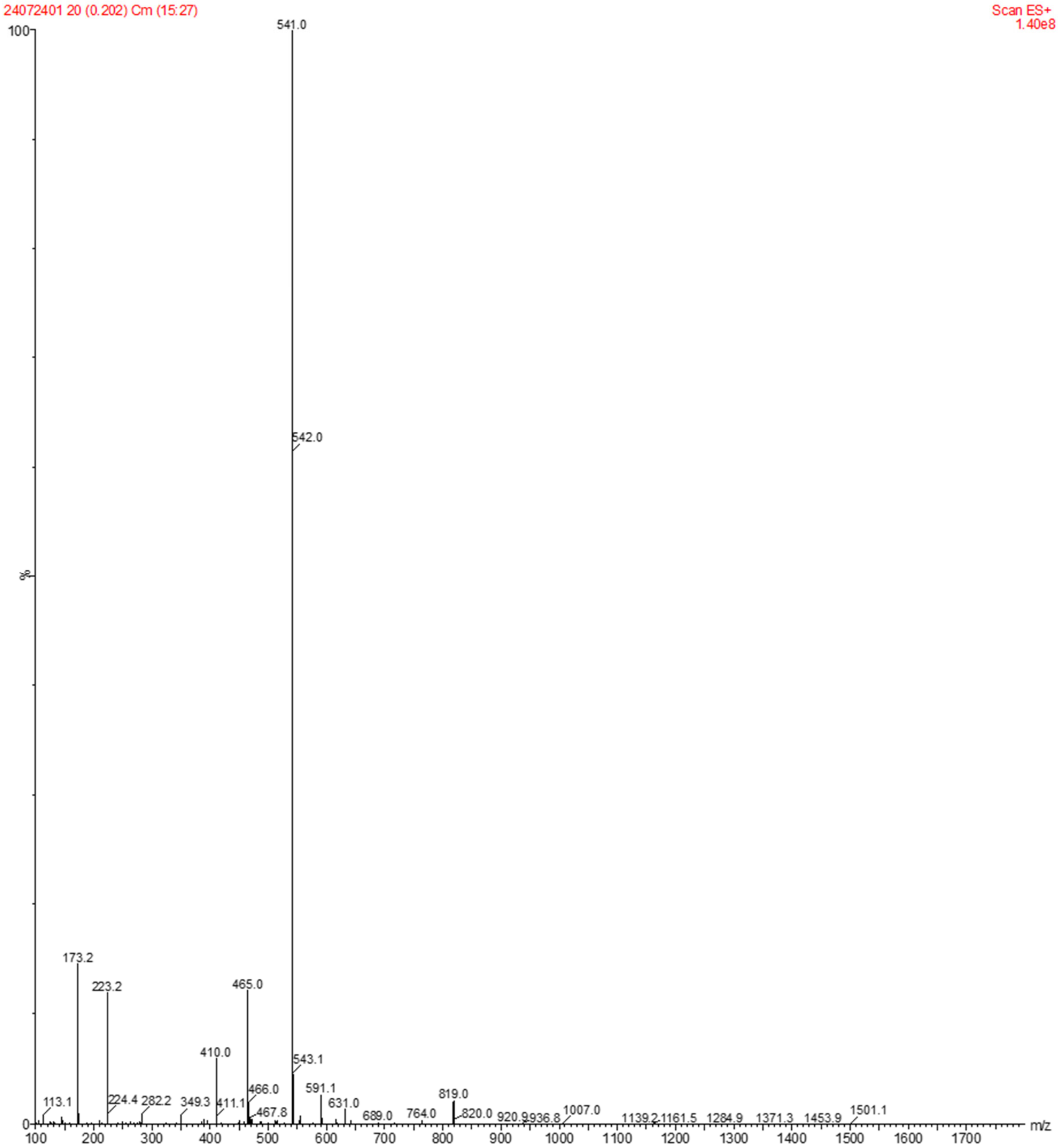

Figure S14. (+)ESI-MS spectrum for compound **4**. Relevant fragments at 541 m/z attributable to $[(NHC^{Bz})_2Au]^+$.

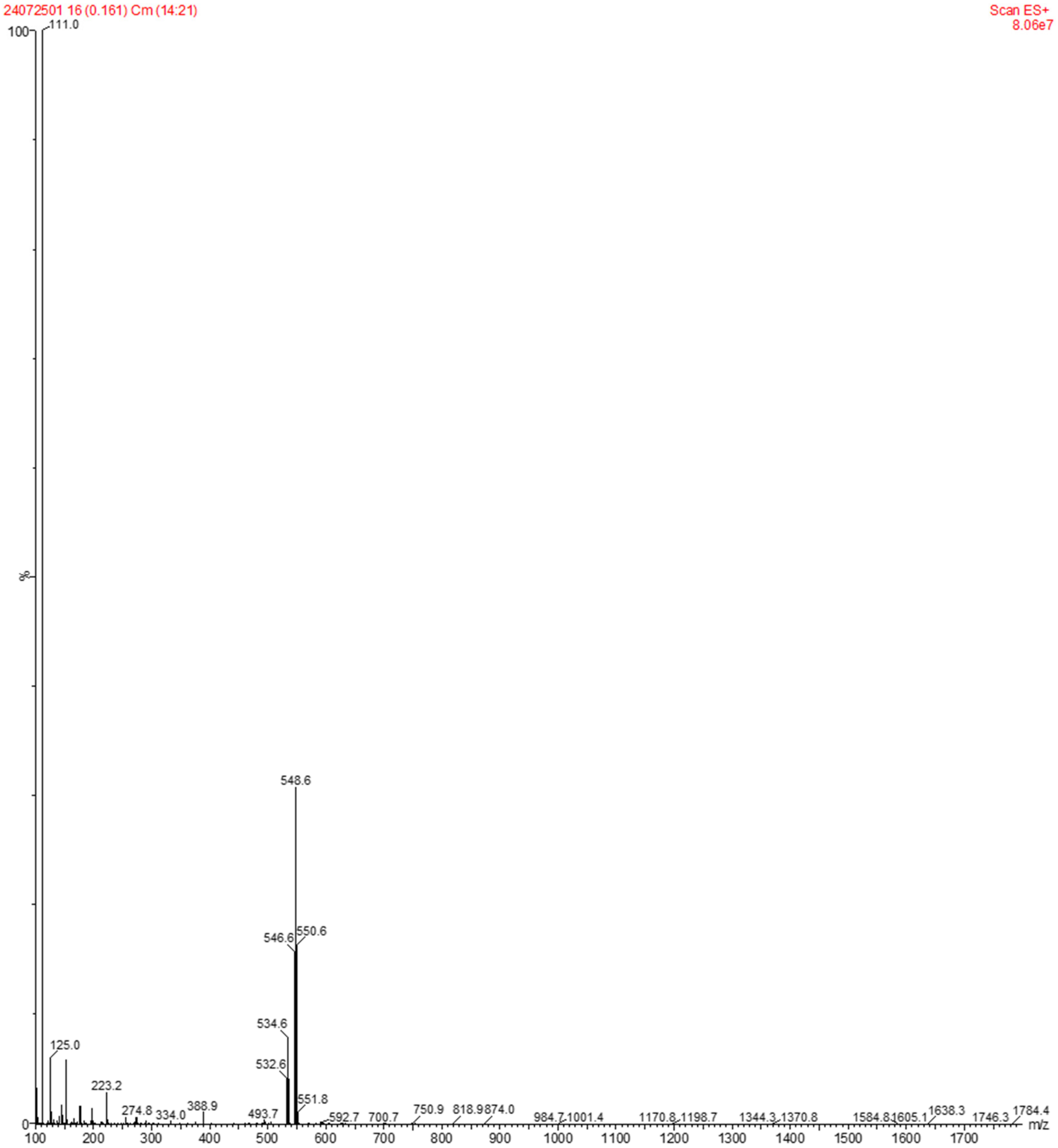

Figure S15. (+)ESI-MS spectrum for compound **5**. Relevant fragments at 549 m/z attributable to $[(NHC^{Me})_2AuBr_2]^+$.

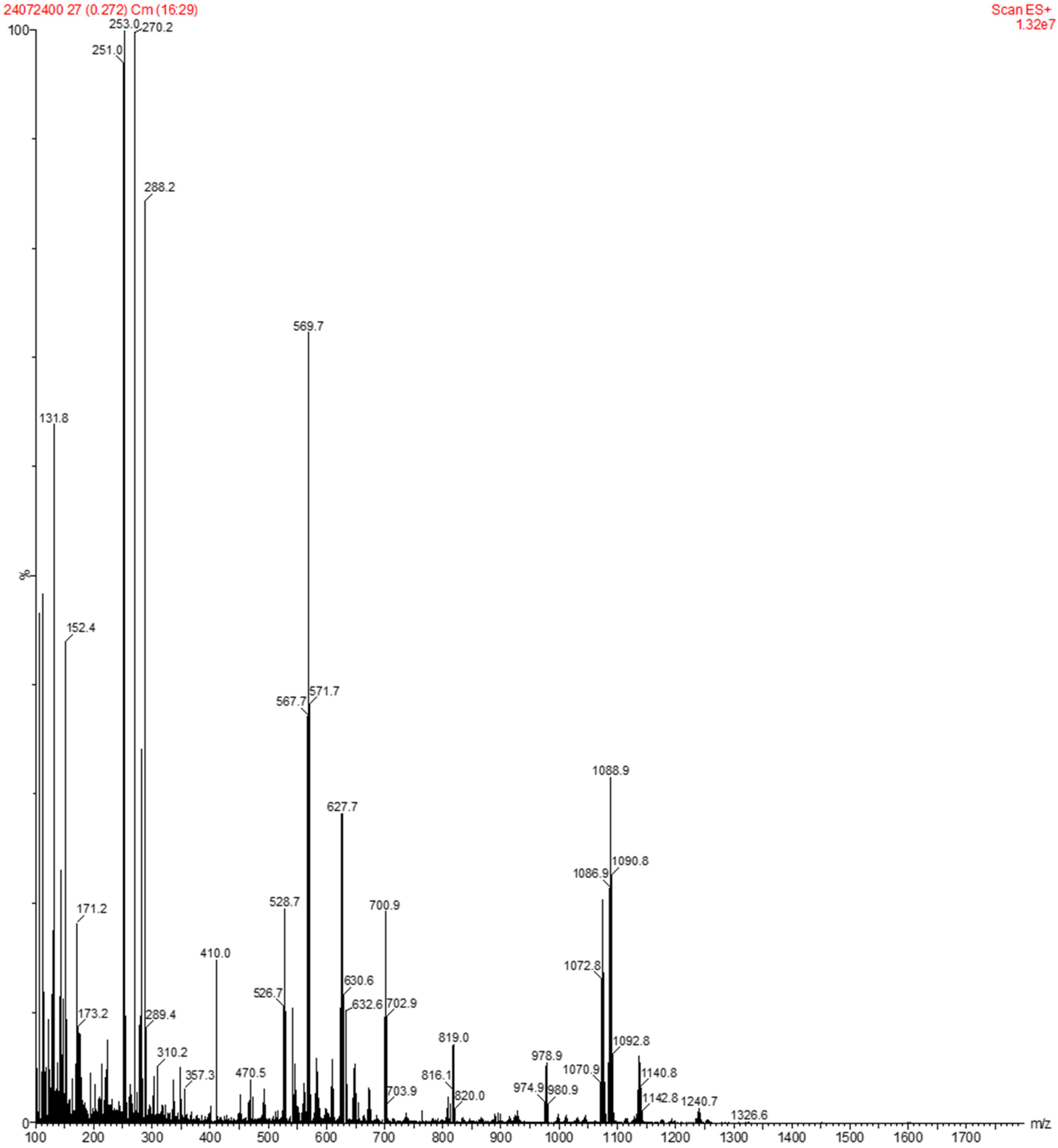

Figure S16. (+)ESI-MS spectrum for compound **6**. Relevant fragments at 528.7 m/z attributable to $[(NHC^{Bz})AuBr_2]^+$, and at 1072.6 m/z attributable to $[(NHC^{Bz})_2Au_2 + Br + 2CH_3CN]^+$.

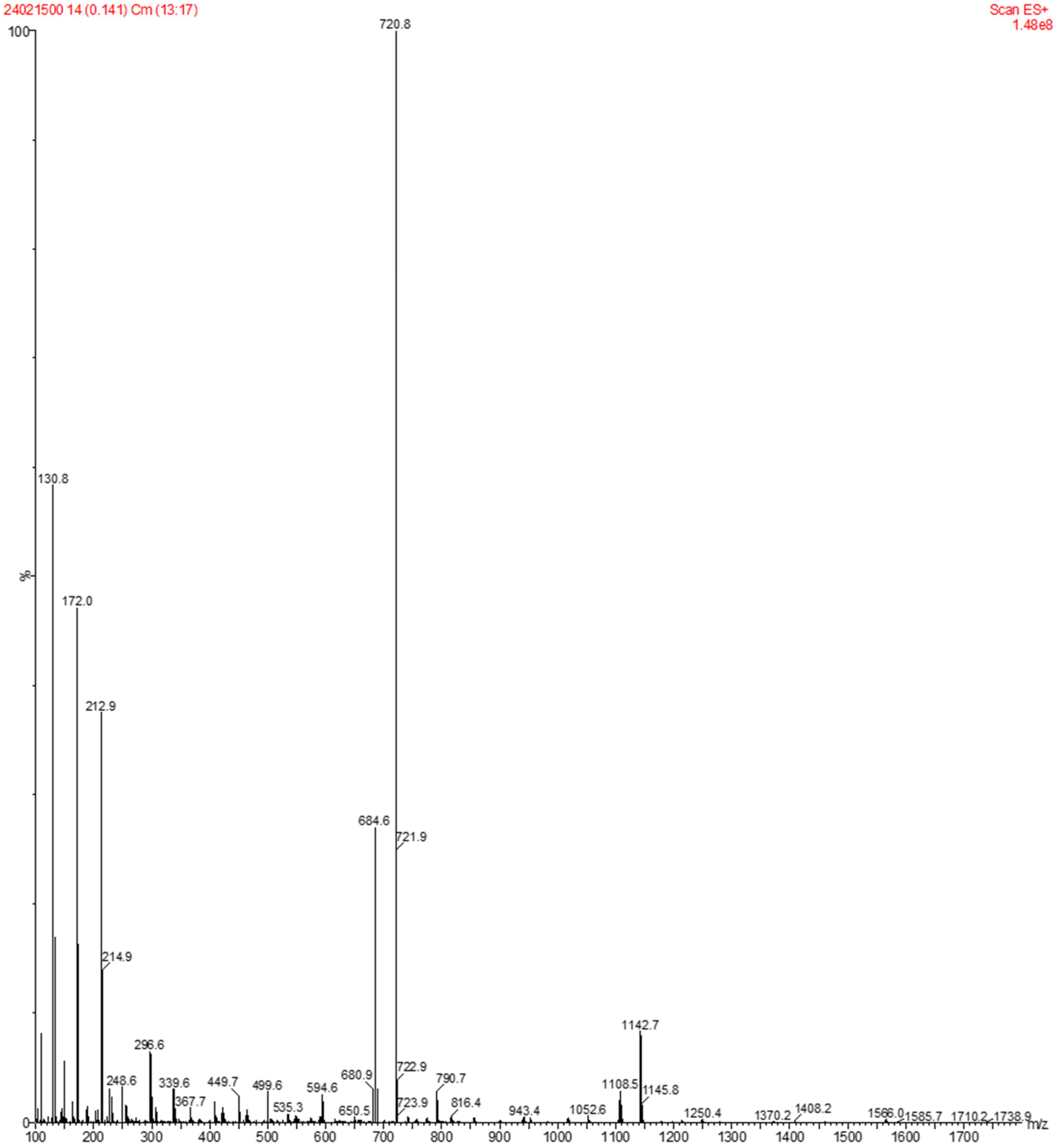

Figure S17. (+)ESI-MS spectrum for compound **7**. Relevant fragments at 684 m/z attributable to $[ImCl_2AuPPh_3]^+$ and at 721.8 m/z attributable to $[(PPh_3)_2Au]^+$.

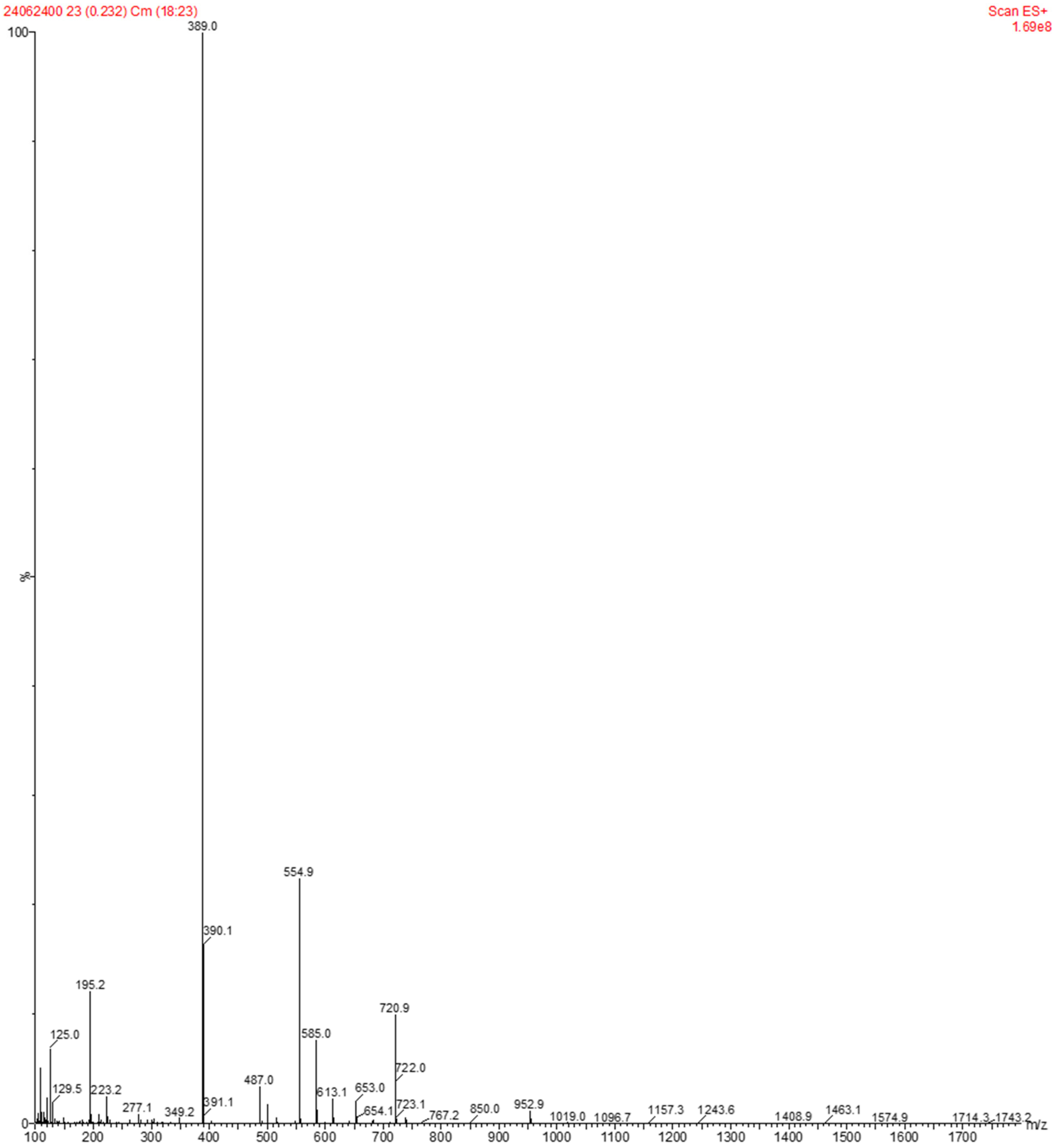

Figure S18. (+)ESI-MS spectrum for compound **8**. Relevant fragments at 554.9 m/z attributable to [NHC^{Me}-AuPPh$_3$]+ and at 721 m/z attributable to [(PPh$_3$)$_2$Au]$^+$.

Stability studies

The stability of the samples 1-8 in DMSO solution was assayed by UV-visible spectroscopy. Spectra were acquired using the Shimadzu UV-2700i spectrophotometer, equipped with the Shimadzu CPS-100 Peltier, at 293 K. Unless diversely specified, 100 μM DMSO solutions of the 1-8 compounds were prepared and spectra were acquired each 10 minutes for a total of 60 minutes. The spectra obtained are reported as following.

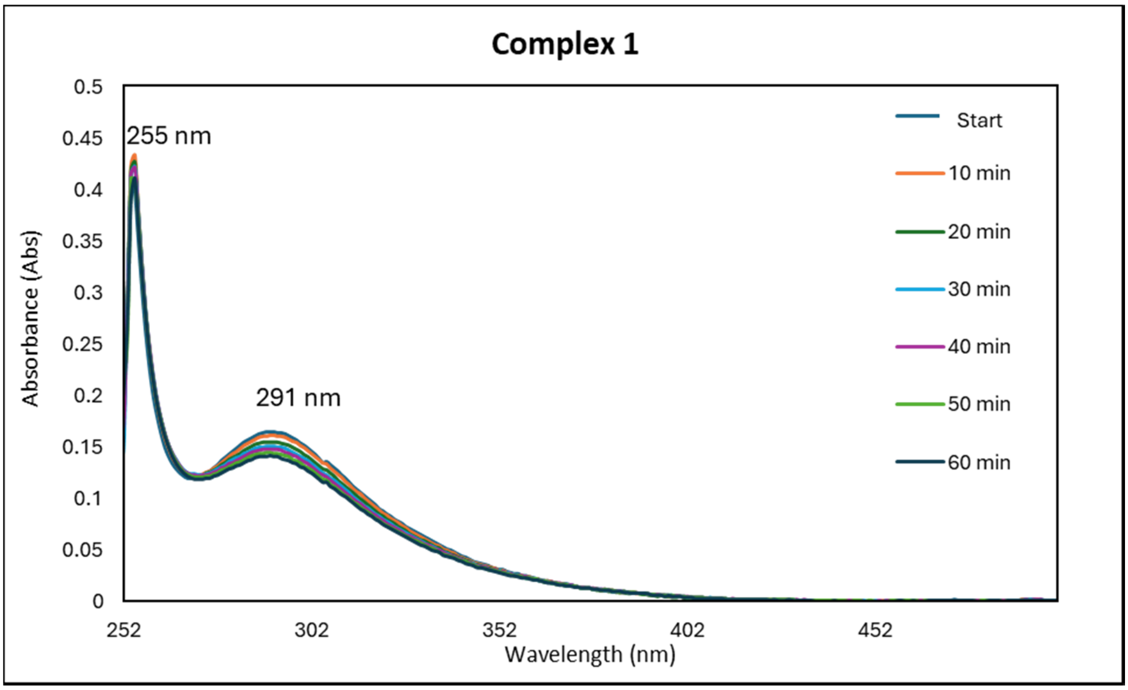

Figure S19. UV-Visible Spectra acquired for complex **1.**

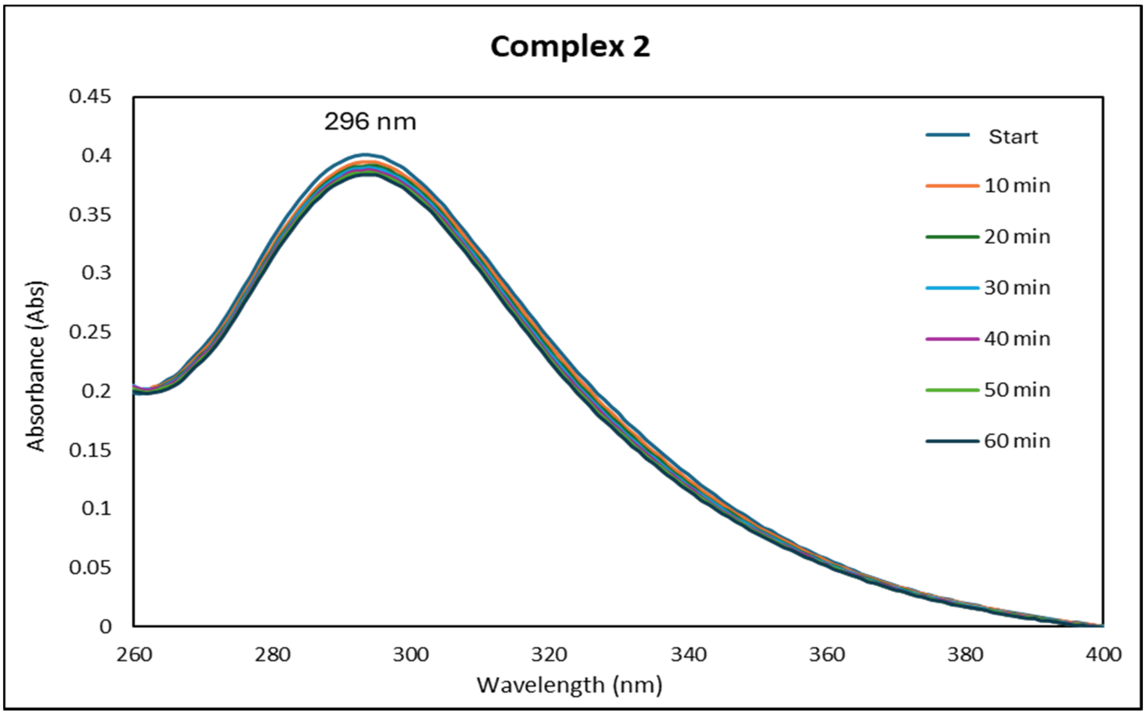

Figure S20. UV-Visible Spectra acquired for complex **2.**

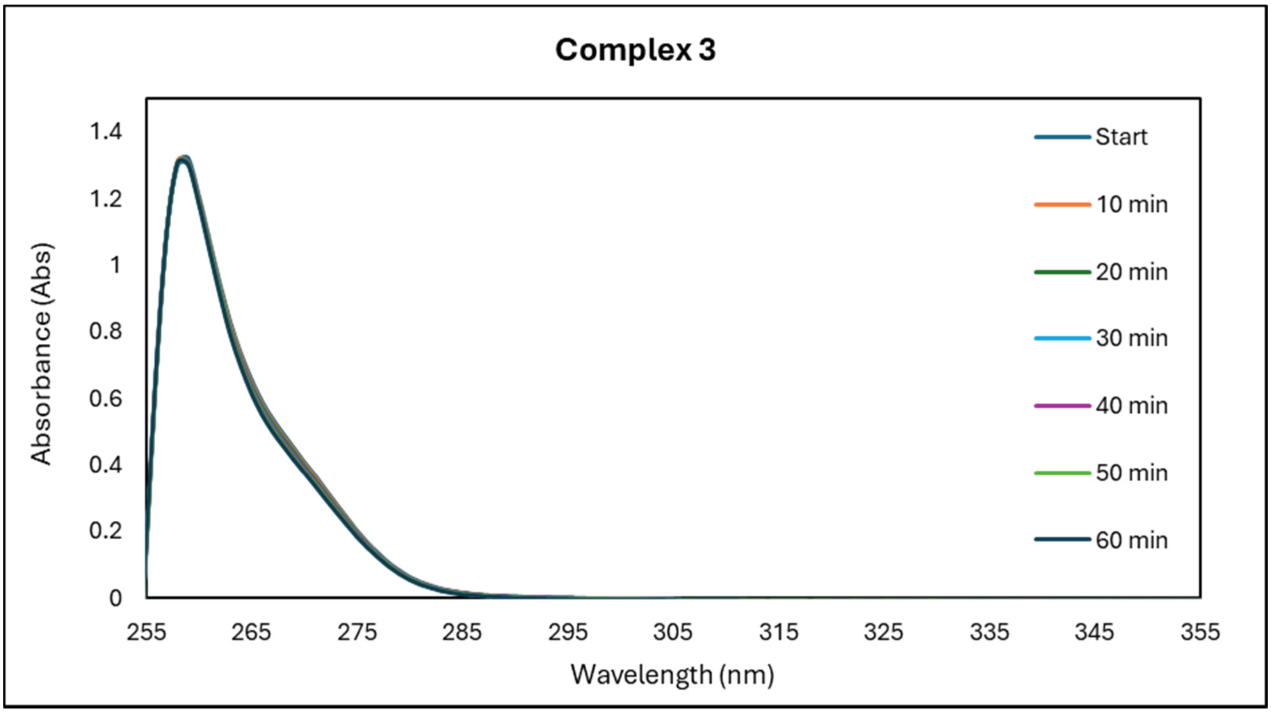

Figure S21. UV-Visible Spectra acquired for complex **3.**

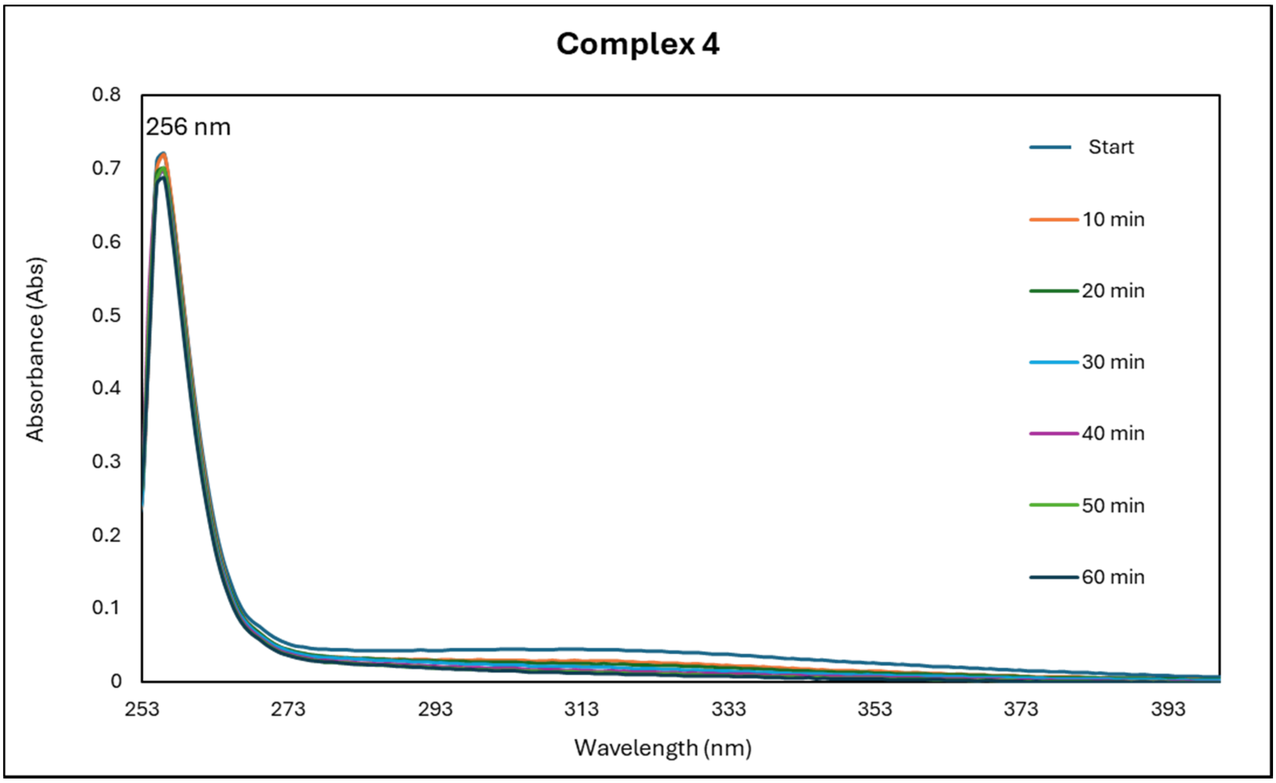

Figure S22. UV-Visible Spectra acquired for complex **4.**

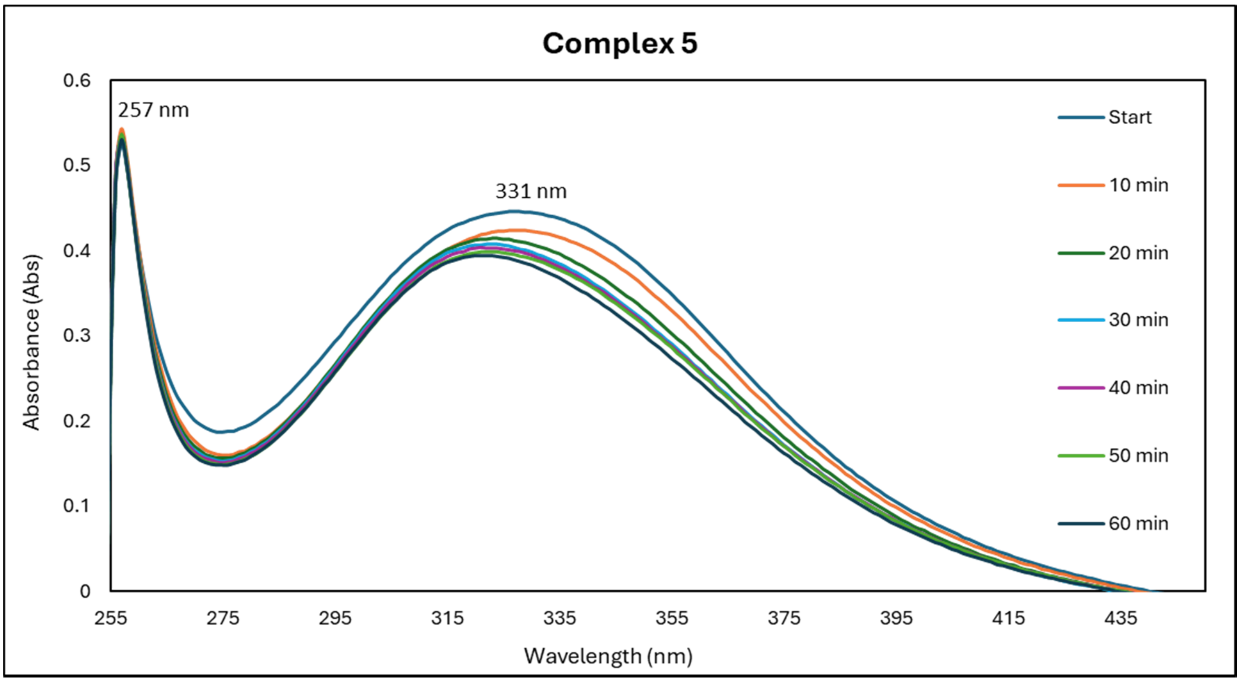

Figure S23. UV-Visible Spectra acquired for complex **5.**

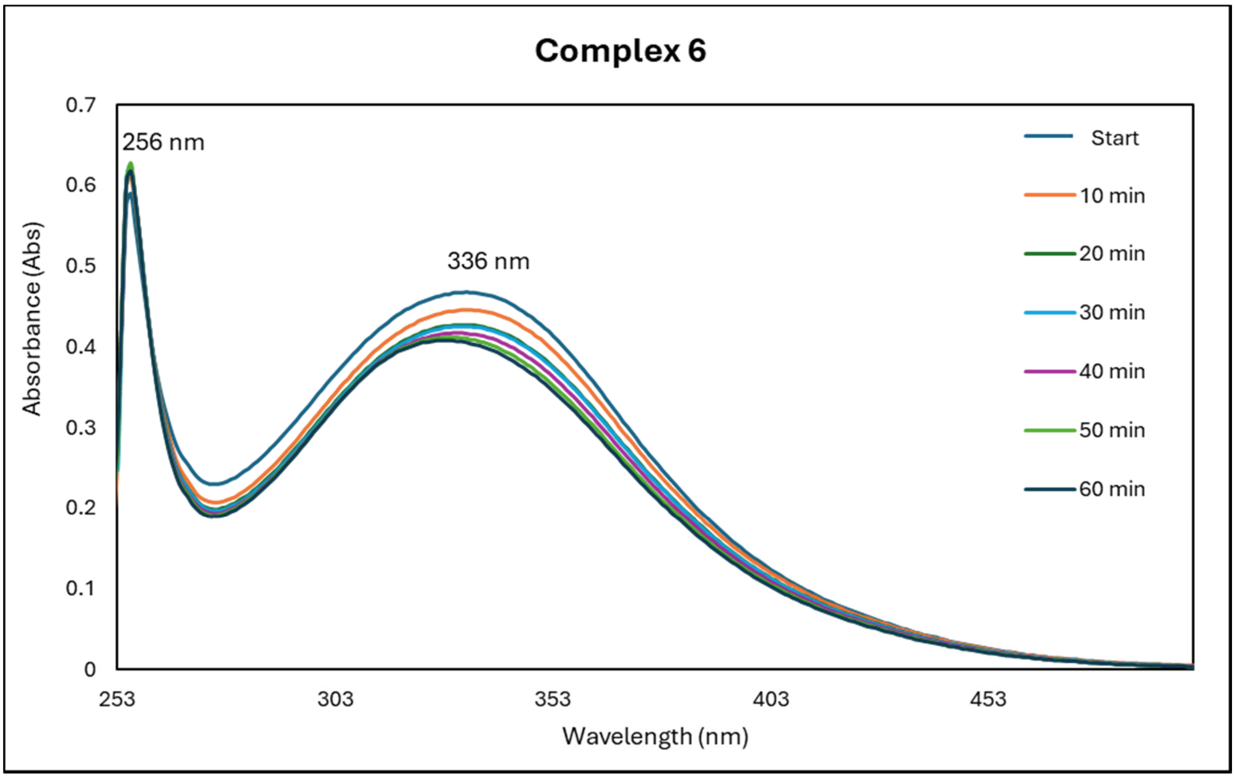

Figure S24. UV-Visible Spectra acquired for complex **6.**

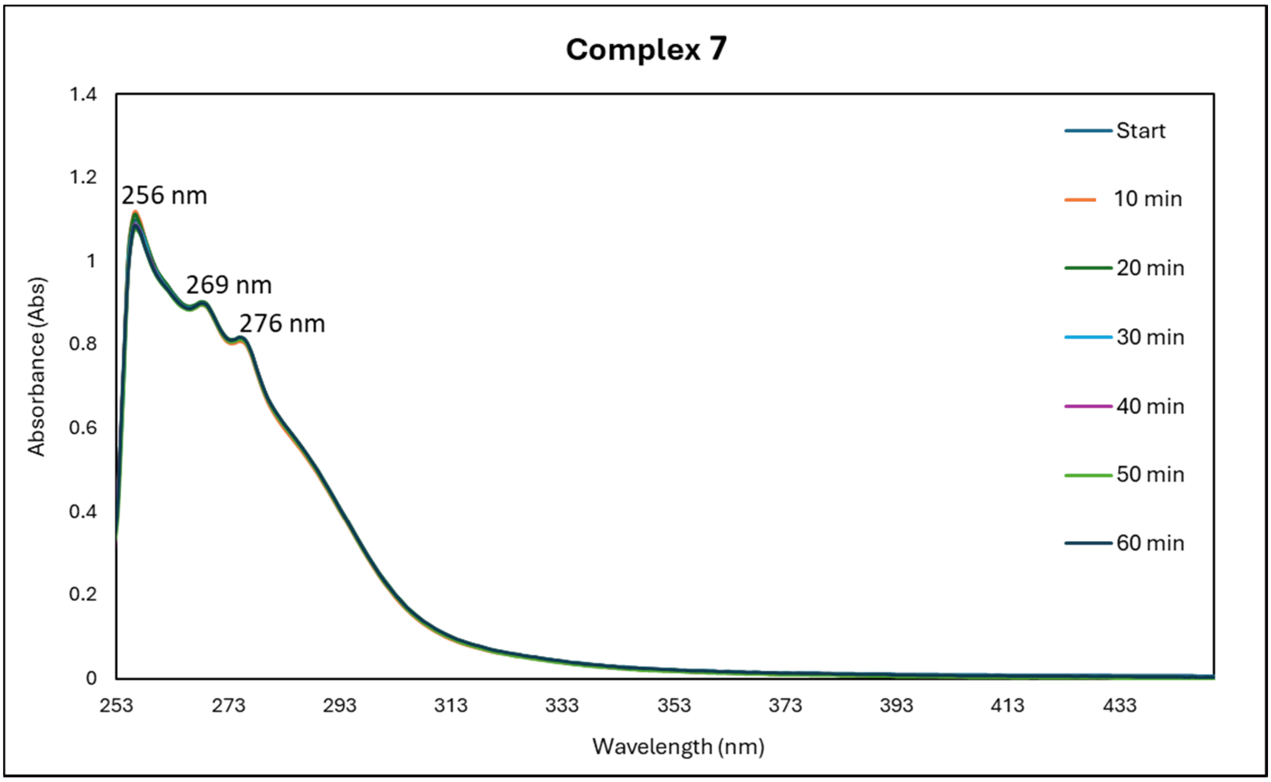

Figure S25. UV-Visible Spectra acquired for complex **7.**

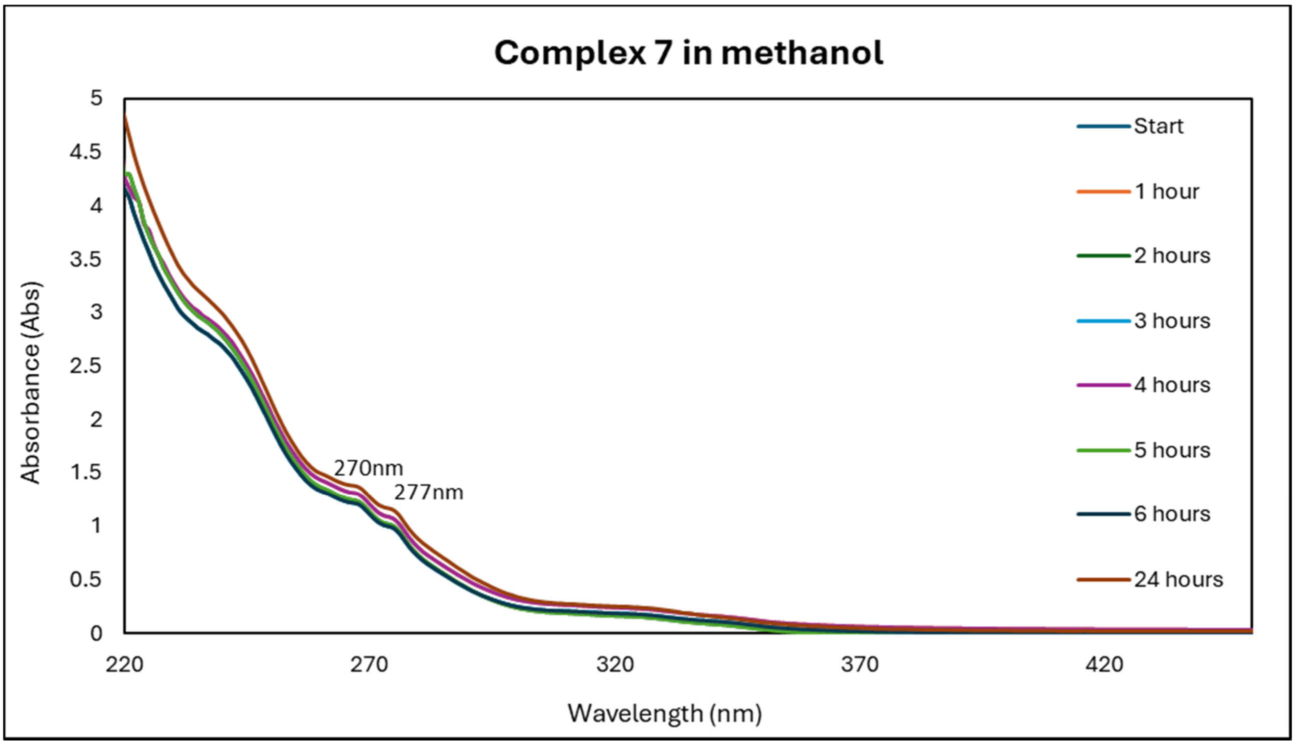

Figure S26. UV-Visible Spectra acquired in 24 hours in methanol for complex **7.**

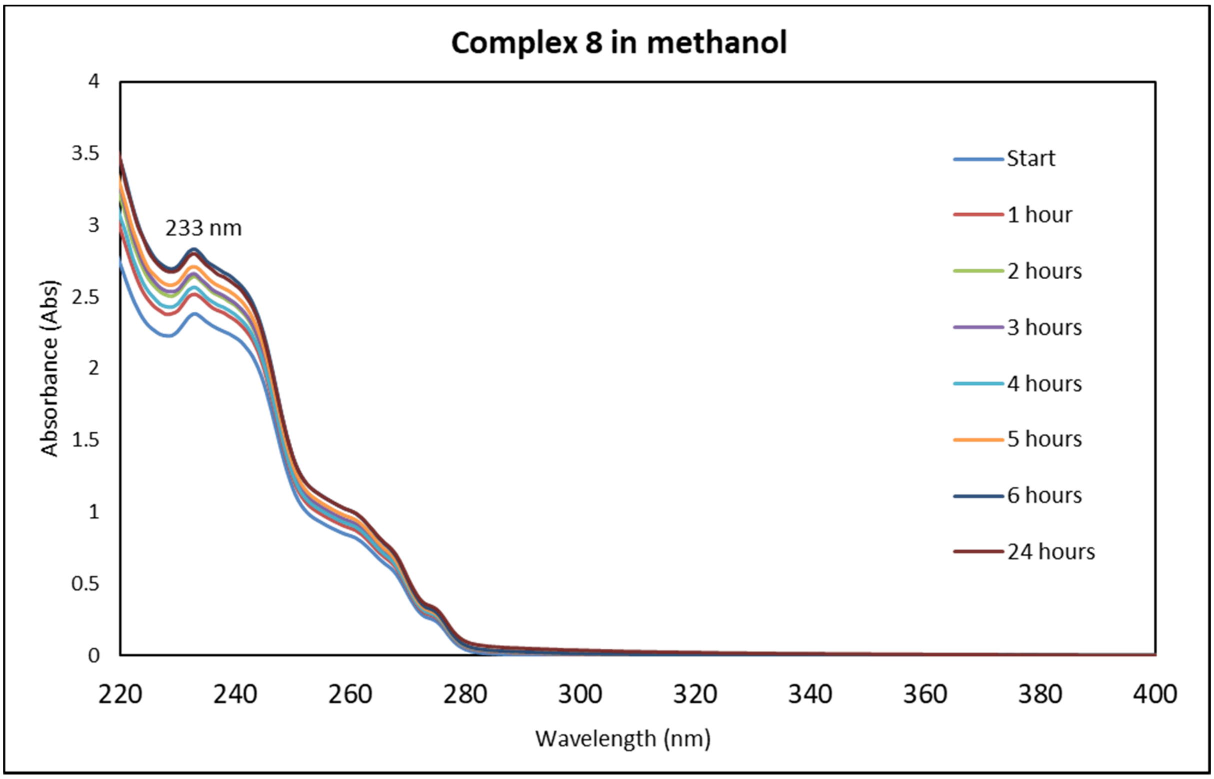

Figure S27. UV-Visible Spectra acquired in 24 hours in methanol for complex **8.**

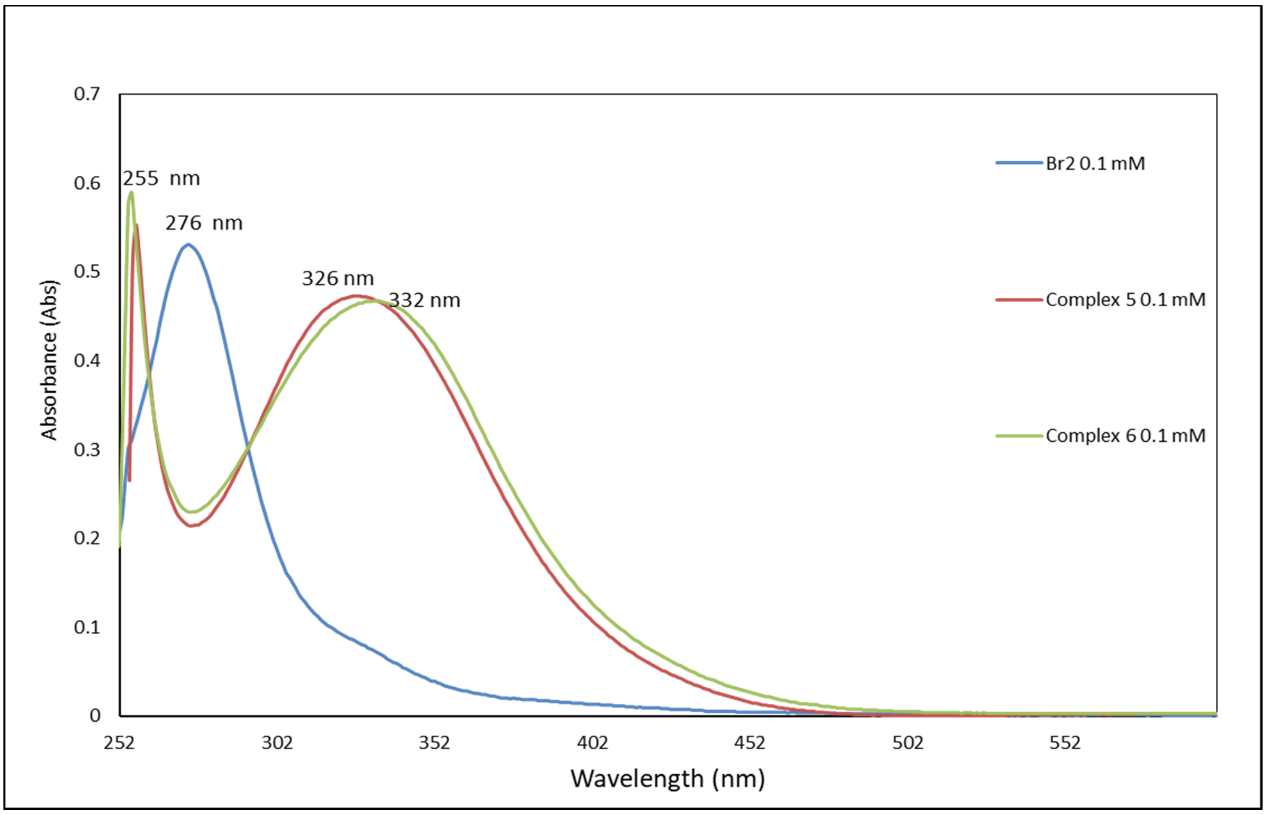

Figure S28. UV-visible spectra of 0.1 mM solutions of compounds 5 and 6 and of bromine in DMSO.

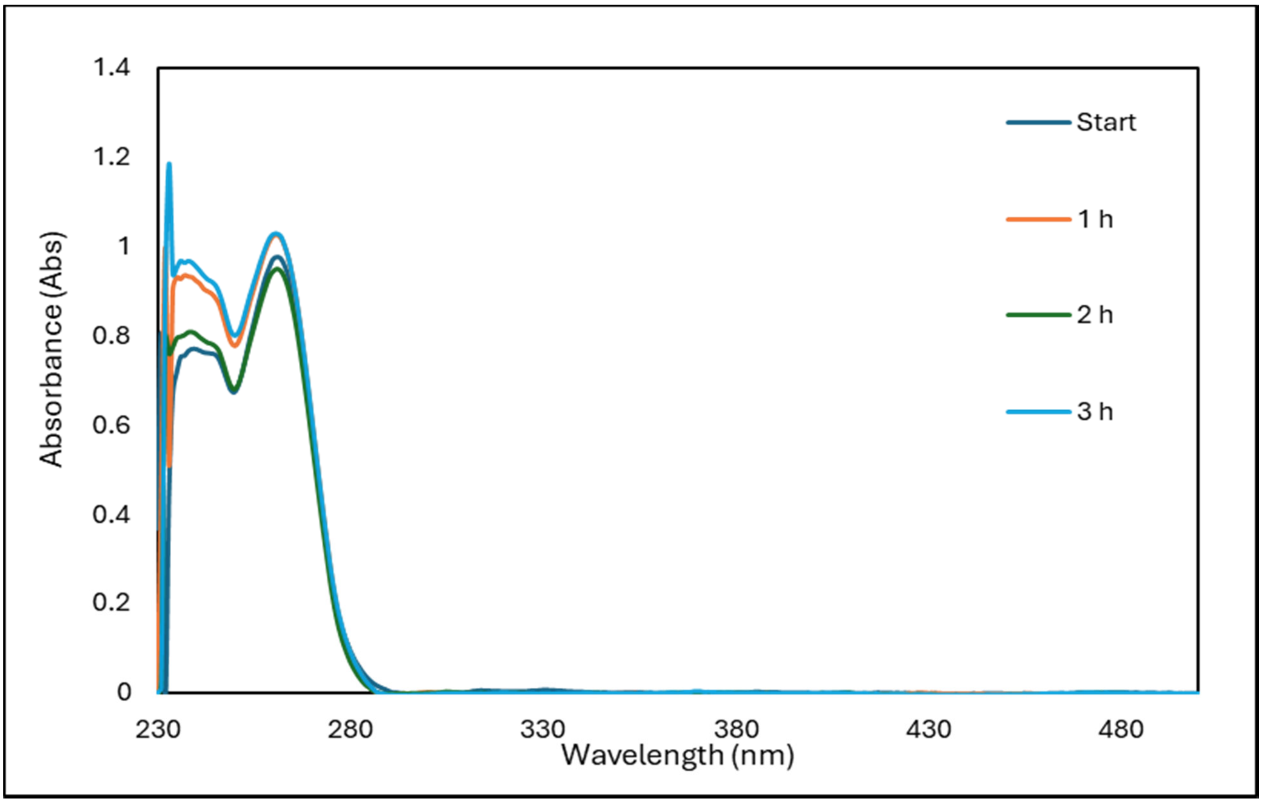

Figure S29. 1 mg of complex 8 was dissolved in 1 mL of DMSO (1.69 mM). The compound was diluted in a solution of phosphate buffer 10 mM (ca) at pH 7.4. UV-Visible spectra were recorded over 3h at 25°C.

Cell Lines Mutations data set

Table S1. Legenda of Table 2S.

**Mutation Data**	
NS	Nonsense substitution
MS	Missense substitution
Del-IF	Deletion - In frame
Del-FS	Deletion - Frameshift
Ins-IF	Insertion - In frame
Ins-FS	Insertion - Frameshift
Del-WG	Whole gene deletion
NS-ext	Nonstop extension
FS	Frameshift
SS	Splice Site Mutation
TSS	Translation Start Site Mutation
NSt	Nonstop Mutation
RNA	RNA Mutation
Som	somatic variant

Possibly som or Likely som	somatic in other cancers
Hom	Homozygous mutation
Het	Heterozygous mutation
Gazdar	Mutation reported by Adi Gazdar (Shigematsu Cancer Res (2005) 65:1642, Shigematsu JNCI (2005) 97:339, Yamamoto Cancer Res (2008) 68:6913)
NCI-Navy	Mutation reported in the original NCI-Navy paper (Phelps J Cell Biochem Suppl (1996) 24:32)
Genes likely involved in cancer (Cancer Gene Census, Sanger)	
Mutated	

Table S2. Lung cancer cell lines mutations data set.

Gene	A549	H157	H460	H1355	H1792	HCC44	H1395	H522	H661	H1993
TP53		[CAG to TAG (stop)](NCI-Navy)	WT	[c.853G>A; p.E285K; MS; hom; som; CLP](COSMIC-V73), [GAG to AAG(miss)](NCI-Navy)	[c.672+1G>A; p.?; hom; som; CLP](COSMIC-V73), [GT to AT(spl don)](NCI-Navy)	[c.128G>T; p.R43L; MS; hom; possibly som; CLP](COSMIC-V73), [c.245G>T; p.R82L; MS; hom; possibly som; CLP](COSMIC-V73), [c.281C>G; p.S94*; NS; hom; possibly som; CLP](COSMIC-V73), [c.524G>T; p.R175L; MS; hom; som; CLP](COSMIC-V73)	WT	[c.175delC; p.P59fs*>32; Del-FS; hom; pred neutral; som](COSMIC-V73), [c.292delC; p.P98fs*56; Del-FS; hom; pred neutral; som](COSMIC-V73), [c.571delC; p.P191fs*56; Del-FS; hom; pred neutral; som](COSMIC-V73), [c.572delC; p.P191fs*56; Del-FS; het; pred neutral; som; CLP](COSMIC-V73), [CCT to CT,?1bp](NCI-Navy)	[AGT to ATT](NCI-Navy), [c.194G>T; p.R65L; MS; het; possibly som; CLP](COSMIC-V73), [c.248G>T; p.S83I; MS; het; possibly som; CLP](COSMIC-V73), [c.365G>T; p.S122I; MS; het; possibly som; CLP](COSMIC-V73), [c.473G>T; p.R158L; MS; het; som; CLP](COSMIC-V73), [c.644G>T; p.S215I; MS; het; som; CLP](COSMIC-V73), [c.77G>T; p.R26L; MS; het; possibly som; CLP](COSMIC-V73)	[c.726C>G; p.C242W; MS; hom; som; CLP](COSMIC-V73), [TGC to TGG](NCI-Navy)
KRAS	[c.34G>A; p.G12S; MS; possibly som](COSMIC-V73), [Mut](Gazdar)	[12CGT](Gazdar), [c.34G>C; p.G12R; MS; som](COSMIC-V73)	[61CAT](Gazdar), [c.183A>T; p.Q61H; MS; som](COSMIC-V73)	[13TGC](Gazdar), [c.37G>T; p.G13C; MS; possibly som](COSMIC-V73)	[12TGT](Gazdar), [c.34G>T; p.G12C; MS; possibly som](COSMIC-V73)	[12TGT](Gazdar), [c.34G>T; p.G12C; MS; hom; som; CLP](COSMIC-V73)	WT	WT	WT	WT
RB1	WT					WT				WT
STK11	[c.109C>T; p.Q37 stop](Gazdar), [c.109C>T; p.Q37*; NS; possibly	[c.ex 2,3 del; p.E98-G155 del., stop at normal but trancated](Gazdar)	[c.109C>T; p.Q37*; NS; hom; possibly som](COSMIC-V73)	[c.143-4 AG>T; p.K48I (fs), stop at 50 aa](Gazdar)	WT		[c.156-169 1G del.; p.E57K (fs), stop at 63 aa](Gazdar), [c.169delG; p.E57fs*7; Del-FS; hom;	WT	WT	[c.595 G>T; p.E199 stop](Gazdar), [c.595G>T; p.E199*; NS; hom; som;

	som](COSMIC-V73)						pred neutral; possibly som](COSMIC-V73)			CLP](COSMIC-V73)
CDKN2A	[c.1_471del471; p.0?; Del-WG; hom; possibly som](COSMIC-V73)	[c.205G>T; p.E69*; NS; hom; possibly som](COSMIC-V73)	WT		[c.330G>A; p.W110*; NS; het; pred neutral; som; CLP](COSMIC-V73)	[c.457+1G>T; p.?; possibly som](COSMIC-V73)				
EGFR	WT	WT	WT		WT	WT	WT	WT	WT	WT
PIK3CA	WT	WT	[c.1633G>A; p.E545K; MS; het; possibly som](COSMIC-V73), [E545K](Gazdar)	WT	WT	WT	WT	WT	WT	WT
PTEN	WT	[c.751G>T; p.G251C; MS; possibly som](COSMIC-V73)	WT					WT		
SMARCA4	[c.2184_2206del23; p.Q729fs*4; Del-FS; hom; pred neutral; possibly som](COSMIC-V73)	WT			WT	WT	[c.809_810delCC; p.P270fs*16; Del-FS; hom; pred neutral; som](COSMIC-V73)			
BRAF	WT	WT	WT	WT	WT	WT	[c.1406G>C; p.G469A; MS; hom; possibly som](COSMIC-V73), [G468A](Gazdar)	WT	WT	WT
NRAS	WT	WT	WT	WT	WT		WT	WT	WT	WT
KDR	WT		[c.3910G>T; p.G1304C; MS; het; som](COSMIC-V73)	WT	WT					
MLL2								WT		
CTNNB1	WT	WT	WT		WT	WT	WT	WT	WT	WT
ATM	WT					WT	[c.7996A>G; p.T2666A; MS; hom; som](COSMIC-V73)			
CREBBP					WT		WT			
NFE2L2	WT									
EP300							WT		WT	WT
NTRK3				WT			WT			WT
PDGFRA							WT			WT
NOTCH2	WT			WT			WT			
APC										
MYH11			[c.2271C>A; p.D757E; MS; het; som](COSMIC-V73), [c.2292C>A; p.D764E; MS; het; som](COSMIC-V73)	WT	WT					
TPR							WT			WT
FUBP1							WT		WT	
WHSC1						WT	WT			
MSH2	[c.965G>A; p.G322D; MS; possibly	WT	WT	[c.1054G>C; p.D352H; MS; het;						

	som](COSMIC-V73)			som](COSMIC-V73)						
ARID1A			[c.6403_6408delATTCTG; p.I2135_L2136del; Del-IF; het; pred neutral; som](COSMIC-V73)	WT	WT					
TNFAIP3			[c.406C>T; p.R136C; MS; het; som](COSMIC-V73)	WT						
U2AF1					WT					
HLF							WT		WT	
KDM6A	WT	WT	WT	WT	WT		WT	WT	WT	WT
SF3B1		WT		WT	WT		WT		WT	WT
HRAS	WT	WT	WT	WT	WT		WT	WT	WT	WT
VHL		WT	WT		WT		WT	WT	WT	WT
MLL3	WT					WT				WT
CARD11				WT			WT		WT	
ROS1							WT			
KIAA1549						WT				WT
MYH9							WT			
PRDM16					[c.1574G>A; p.R525Q; MS; het; possibly som; CLP](COSMIC-V73)	WT		WT		
IKZF1							WT		WT	
NUP214			WT			WT	[c.4738G>T; p.E1580*; NS; het; som](COSMIC-V73)			
CBLB							WT			
PIK3R1	WT		WT				WT	WT		
RET						WT	WT	WT		WT
FBXW7			WT				WT			
NOTCH1	WT			WT			WT		WT	
GNAS							WT			
NUMA1							WT			
DNMT3A							WT			
RAD21			[c.353A>T; p.D118V; MS; het; som](COSMIC-V73)	WT						
CDH1							WT			
ARID2			WT	WT				WT		
RALGDS							WT			
POT1							WT			
GOLGA5			[c.595A>G; p.K199E; MS; het; som](COSMIC-V73)	WT		WT				
NACA				WT			WT			
RAF1							WT			
GPC3							[c.490G>C; p.D164H; MS; hom; som](COSMIC-V73)			
BCOR				[c.377C>T; p.P126L; MS; hom; possibly som; CLP](COSMIC-V73)	WT	[c.2266G>T; p.E756*; NS; hom; som](COSMIC-V73)				
ZNF521							WT		WT	

SETD2							WT			
PML							WT			
PALB2							WT			
RNF43										
ERBB2	WT	WT	WT	WT	WT	WT	WT	WT	WT	WT
EML4	WT	WT	WT	WT	WT	WT	WT		WT	WT
MAX	WT		WT			WT	WT	WT	WT	WT
NF2		WT	WT		WT		WT	WT	WT	WT
TRRAP					WT		WT			
MET	WT		WT				WT	WT		WT
MAP2K1	WT				WT		WT	WT		WT
PTPRC							WT			
ARHGEF12	WT		WT				WT			
HIP1	[c.830G>A; p.S277N; MS; het; som](COSMIC-V73)			WT						
FANCD2							WT			
NUP98					WT		WT			
PDGFRB							WT			
KDM5A							WT			
WRN							WT			
TSHR			WT			WT				WT
PDE4DIP			WT				WT		WT	
MEN1										
FGFR2						WT	WT			
USP6							WT			WT
CDH11			WT				WT	WT	WT	
CBLC							WT			
TRIP11	WT						WT	[c.3083G>A; p.R1028Q; MS; het; som](COSMIC-V73)	WT	
ATRX							WT		WT	WT
PICALM							WT	WT		WT
ETV1						WT	WT		WT	
LRIG3					WT		WT			
MLH1					[c.52C>T; p.R18C; MS; het; possibly som; CLP](COSMIC-V73)	WT				
NSD1										
ABL2						WT	WT			
XPO1							WT			
DICER1							WT			
TCF12							WT			
NTRK1							WT			
PPP2R1A	WT		[c.192G>T; p.E64D; MS; hom; som](COSMIC-V73)	WT						
LIFR			[c.1477C>T; p.L493F; MS; het; pred neutral; som](COSMIC-V73)	WT	WT					
TRIM33							WT			
TCEA1							WT			
BUB1B							WT		WT	WT
LCP1							WT	[c.1412C>A; p.A471E; MS; hom; som](COSMIC-V73)		
COL1A1							WT		WT	
PTPN11							WT			
EBF1										
ITK							WT			

IDH1							WT	WT		
ATIC						WT	WT			
NCOA2						WT	WT			
ELN					WT				WT	
TRAF7							WT			
SUZ12							WT			
MYB							WT			
BRIP1							WT			
FBXO11							WT			
NCOA1							WT			
FOXP1						WT	WT			
MAP2K4							WT			
ATP2B3						WT	WT			
LPP							WT			
TERT	WT						WT			
MSH6							WT			
EZH2			WT				WT			
THRAP3							WT		WT	
KDM5C							WT			
STAT5B							WT			
FGFR3				WT			WT			
MLLT3							WT		WT	
PCSK7							WT	WT		
TMPRSS2							WT			
ERG							WT			
MLLT1							WT			
PBRM1										
IL7R										
RUNX1						WT	WT			
EIF4A2							WT			
GMPS							WT			
BMPR1A							WT			
FH	[c.1391G>T; p.G464V; MS; het; som](COSMIC-V73)			WT						
BCL3							WT			
EXT1							WT			
CAMTA1										
FANCC				WT			WT			
MLF1							WT			
ETV4							WT			
GATA2							WT			
WIF1							WT			
HOOK3										
HNRNPA2B1						WT			WT	
ELF4							WT			
SUFU	[c.1232C>T; p.T411M; MS; het; possibly som; CLP](COSMIC-V73)			WT						
PDGFB							WT			
RARA							WT			
DDX5							WT			
LHFP							WT			
TSC1										
CCND1							WT			
SOCS1							WT			
NR4A3										
CDC73										
C15orf55			[c.1001C>A; p.P334Q; MS; hom; som](COSMIC-V73)	WT						
TET2										
FAM46C										
SDC4										
SETBP1	WT		WT	WT		WT	WT	WT	WT	WT
AKT1	WT	WT	WT			WT	WT	WT	WT	

FCGR2B							WT			
CLTCL1				WT			WT			
AKAP9					WT		WT		WT	
FANCA							WT			WT
MAP2K2	WT		WT		WT		WT	WT		WT
KTN1							WT	WT		
NF1			WT	WT			WT			
ALK							WT			
MLLT4							WT			
SMARCB1	WT	WT					WT			
CARS							WT			WT
BCR						WT	WT			
PCM1										
PER1							WT			
JAK2							WT			WT
BRCA2							WT	WT		
CBFA2T3						WT	WT			WT
NFIB						WT	WT			
GPHN							WT			
TSC2			WT							
DDX10							WT			
GAS7			WT				WT			
AXIN1						WT	WT			
ERCC2				WT			WT		WT	
CHN1						WT	WT			
FLT3							WT			
WHSC1L1							WT		WT	
EPS15				WT			WT	WT	WT	
KIT							WT			WT
ERCC3				WT			WT			
CACNA1D	WT								WT	
SRGAP3							WT			
CHEK2	WT		WT				WT		WT	
BLM							WT			
MED12	WT						WT			
RANBP17				WT						WT
TCF3	WT		WT				WT			
TOP1				WT			WT			
BCL6			WT				WT			
PBX1							WT			
STAG2							WT			
BCL11A							WT			
PMS2							WT			
PAX5							WT			
NPM1							WT			
NIN							WT			
CIITA							WT			
BRCA1							WT			
STAT3							WT			
BRD4				WT			WT			WT
MLLT10				WT			WT			
ARNT							WT			
PMS1			WT				WT			
CLTC				WT			WT			WT
ETV6							WT			WT
FIP1L1							WT			
ERCC5							WT			
MLL										
JAK3				WT			WT			
ABL1							WT			
SYK							WT	WT		
CNOT3										
MDM4	WT						WT			
ERCC4							WT			
CDKN2C	WT						WT			
DDX6							WT			WT
BCL9				WT			WT			
BRD3							WT			
IRF4							WT			
IL6ST							WT			
TFRC							WT			
SH3GL1							WT			

ABI1							WT			
MPL							WT			
PAX3							WT			
CDK6	WT		WT				WT			
RAP1GDS1							WT			
MAML2							WT			
CBL							WT			
MALT1						WT	WT			
FUS				WT			WT			
ECT2L										
FLI1					WT		WT			
GNAQ							WT			
DEK							WT			
SFPQ							WT			
FOXL2	WT		WT				WT	WT		
HMGA2							WT			
JAK1							WT	WT		
GATA3							WT			
RAC1							WT			
XPC	WT						WT			
MSN							WT			
MSI2					WT		WT			
PAX8							WT			WT
WAS							WT			WT
FGFR1OP							WT			
TRIM27							WT			
BCL7A							WT			
PRDM1							WT			
IL21R							WT			
DAXX							WT			
RECQL4							WT			
FANCE							WT			
BIRC3							WT			
SMARCE1							WT			
TFPT							WT			
AKT2							WT			
TPM3							WT			
DDB2							WT	WT		
TCL1A							WT			
CASP8							WT			
ETV5							WT		WT	
PRCC							WT			
ASPSCR1							WT			
SET							WT			
ACSL3							WT			
ZRSR2							WT			
KIF5B										
SMO									WT	
PAX7							WT			
LCK						WT	WT			
BCL11B							WT			
POU2AF1							WT			
MUTYH				WT			WT			
CCND3	WT						WT			
CDK12							WT			
ZNF384							WT			
MDM2							WT			
KLK2							WT			
GOPC							WT			
JAZF1							WT			
NDRG1							WT			
CCNE1							WT			
SRSF2				WT			WT			
TNFRSF14				WT			WT			
SEPT6							WT			
ZNF331							WT			
ALDH2					WT		WT			
IDH2							WT			
GNA11						WT	WT			
ELL							WT			
TPM4							WT			
PRKAR1A							WT			

SS18L1				WT	
TTL				WT	
SS18				WT	
PAFAH1B2				WT	
CCND2				WT	
PHF6				WT	
TFG				WT	
CDK4	WT	WT		WT	
CD274				WT	
CCDC6				WT	
XPA		WT		WT	
SDHC				WT	
HEY1				WT	
BCL2				WT	
FANCG				WT	
CBFB				WT	
RPN1				WT	
HOXA11				WT	
TFEB				WT	
TAL1				WT	
ELK4				WT	
HOXD13				WT	
RPL22				WT	
TFE3				WT	
DNM2					WT
MYCN				WT	
RBM15				WT	
TLX3				WT	
KLF4				WT	
PLAG1				WT	
NT5C2					
REL				WT	WT
FOXA1				WT	
LMO1				WT	
GATA1				WT	
BCL10				WT	
FANCF				WT	
SDHB				WT	
TNFRSF17				WT	
IL2				WT	
MDS2				WT	
LMO2				WT	
HOXC11				WT	
BTG1				WT	
WT1					
CYLD					
FGFR1				WT	
CRTC3					
MUC1				WT	
SLC34A2					
TIF1				WT	
NONO				WT	
YWHAE				WT	
MAFB				WT	
CDX2				WT	
RPL10				WT	
TLX1				WT	
FSTL3				WT	
ATF1				WT	
MDS1				WT	
HOXC13				WT	
MYC				WT	
OLIG2				WT	
NKX2-1				WT	
HOXA13				WT	
HOXD11				WT	
P2RY8				WT	
MAF				WT	
PIM1				WT	
HOXA9				WT	
DDIT3				WT	
ATP1A1					

CHIC2		WT	
LYL1		WT	
CCNB1IP1		WT	
MTCP1		WT	
SOX2		WT	
SDHD		WT	
POU5F1		WT	
HMGA1		WT	
COX6C		WT	
OMD		WT	
FEV		WT	
JUN		WT	
TCL6		WT	
TAF15		WT	
BAP1			
CSF3R			
CIC			
TCF7L2			WT
ASXL1			
CREB3L2			
MITF			
SSX1	WT		
CREB3L1			WT
EXT2			
SBDS			WT
MKL1			
RUNDC2A			
WWTR1			
VTI1A			
PPARG			
EWSR1			
FNBP1			
EZR			
PHOX2B			
CD79B			
NFKB2			
LASP1			WT
NCOA4			
MN1			
CREB1			
HERPUD1			
KCNJ5			
CD74			
SLC45A3			
MYCL1			
CALR			
ZNF198			
FAM22A			
RPL5			
ZNF278			
SH2B3			
MYD88			
FHIT			WT
C2orf44			
PRF1			
CHCHD7			
CD79A			
H3F3A			
CANT1			
H3F3B			
TAL2			
HIST1H3B			
HIST1H4I			
CEBPA			
CRLF2			
